# Correlation‐adjusted regression survival scores for high‐dimensional variable selection

**DOI:** 10.1002/sim.8116

**Published:** 2019-02-22

**Authors:** Thomas Welchowski, Verena Zuber, Matthias Schmid

**Affiliations:** ^1^ Department of Medical Biometry, Informatics and Epidemiology University Hospital Bonn Bonn Germany; ^2^ MRC Biostatistics Unit Cambridge University Cambridgeshire UK; ^3^ Department of Epidemiology and Biostatistics Imperial College London London UK

**Keywords:** biomarker discovery, breast cancer, multigene signature, personalized medicine, prostate cancer, survival modeling

## Abstract

**Background:** The development of classification methods for personalized medicine is highly dependent on the identification of predictive genetic markers. In survival analysis, it is often necessary to discriminate between influential and noninfluential markers. It is common to perform univariate screening using Cox scores, which quantify the associations between survival and each of the markers to provide a ranking. Since Cox scores do not account for dependencies between the markers, their use is suboptimal in the presence of highly correlated markers.

**Methods:** As an alternative to the Cox score, we propose the correlation‐adjusted regression survival (CARS) score for right‐censored survival outcomes. By removing the correlations between the markers, the CARS score quantifies the associations between the outcome and the set of “decorrelated” marker values. Estimation of the scores is based on inverse probability weighting, which is applied to log‐transformed event times. For high‐dimensional data, estimation is based on shrinkage techniques.

**Results:** The consistency of the CARS score is proven under mild regularity conditions. In simulations with high correlations, survival models based on CARS score rankings achieved higher areas under the precision‐recall curve than competing methods. Two example applications on prostate and breast cancer confirmed these results. CARS scores are implemented in the R package carSurv.

**Conclusions:** In research applications involving high‐dimensional genetic data, the use of CARS scores for marker selection is a favorable alternative to Cox scores even when correlations between covariates are low. Having a straightforward interpretation and low computational requirements, CARS scores are an easy‐to‐use screening tool in personalized medicine research.

## 1 INTRODUCTION

One of the key issues in personalized medicine is to identify genetic marker signatures for the planning and the prognosis of targeted cancer therapies. With more than one of three people developing some form of cancer during their lifetimes,^1^ individualized therapies based on genetic markers are expected to play a major role in improving progression‐free and overall survival of cancer patients. Among men, for example, prostate cancer is the cancer with the highest prevalence. While there are several clinical models available for predicting disease progression, it remains a challenging task to develop molecular signatures and improve predictive accuracy of existing models.^2^

Since cancer research is heavily focused on time‐to‐event outcomes such as progression‐free survival, metastasis‐free survival, and/or overall survival, survival analysis is one of the predominant statistical approaches to analyze data collected in clinical cancer trials. When the aim is to relate a time‐to‐event outcome to a set of predictors (eg, clinical information or genetic markers), it is common to use a survival model such as the proportional hazards model by Cox.^3^ However, when data are high dimensional (eg, when the number of measured genetic markers exceeds the sample size), it is impossible to fit a Cox regression model including all available covariates. A solution to this problem could be to use regularized methods such as ridge‐penalized Cox regression, but even these methods often break down when the number of available markers (in particular, the number of noninfluential markers) is large. It is therefore common practice to carry out data‐driven variable selection before fitting the survival model and to include only those “influential markers” that have passed the selection step.

The predominant method for variable selection in cancer research is univariate screening, which evaluates the associations between the outcome of a trial and each covariate separately, eg, by computing correlation coefficients or fitting simple regression models. The coefficients of association are usually ranked by magnitude, and the most highly ranked covariates are selected for inclusion in the statistical model of interest. Fan and Lv^4^ have provided a theoretical justification for this approach by showing that univariate screening is suitable for identifying influential covariates with high probabilities under mild regularity conditions. Still, a major problem of this approach is that associations between covariates are ignored. For example, the set of selected markers may include so‐called “antagonistic” variables that are highly correlated among each other but whose correlation/regression coefficients have opposing signs. While decreasing the robustness of the final statistical model, such marker variables will also cause influential markers with weaker univariate associations to be dropped from the model. This information loss is particularly problematic when the number of selected markers needs to be restricted to a small value due to sample size or cost limitations.

In survival analysis with a right‐censored time‐to‐event outcome, univariate screening is mostly done by computing Cox scores, which are given by either the Z scores obtained from univariate Cox regression models or by the p values obtained from the respective likelihood ratio or score tests. Although p values can be corrected for multiple testing (eg, see the work of Benjamini and Hochberg^5^) to identify informative covariates, Cox scores share the same disadvantages as the univariate screening methods mentioned above.

To address these problems, we consider the correlation‐adjusted regression (CAR) score approach, which provides a criterion for variable ranking that is based on the decorrelation of covariates in linear regression. By applying a Mahalanobis‐type “decorrelating” transformation to the covariates, CAR scores measure the correlations between the decorrelated variables and the continuous outcome. The set of correlation coefficients defines a ranking of the covariates, which can be used to select informative variables in the same way as with the univariate screening methods described above. As the correlation coefficients among the covariates tend to zero, CAR scores become identical to the correlations between the nontransformed covariates and the outcome. In simulations for linear regression, CAR scores outperformed methods for regularized regression (boosting, lasso) with regard to their ability to correctly recover causal genetic markers and their rankings.^6^ On the other hand, CAR scores have not been used to model time‐to‐event outcomes in cancer research, as an extension of the CAR approach to right‐censored data has been lacking so far.

The purpose of this paper is therefore to develop a CAR‐based method for ranking high‐dimensional sets of genetic marker variables in survival analysis. The resulting score, which, in the following, will be termed correlation‐adjusted regression survival (CARS) score, quantifies the correlations between the log‐transformed survival time Y=log(T) and the decorrelated set of covariates **X**. We will first define a theoretical version of the CARS score on the population level (Section 2.1). Afterward, we will provide details on the estimation of the scores from a sample of right‐censored data (Section 2.2). Specifically, we will show that all relevant expressions can be estimated using inverse‐probability‐of‐censoring (IPC) weighting techniques, as proposed by Van der Laan and Robins.^7^ In Section 3, we will present the results of a simulation study that was carried out to compare the CARS approach to univariate screening based on Cox scores. In addition, we will apply CARS scores to the Swedish Watchful Waiting Cohort data^2^ and to a data set on invasive breast cancer.^8^ The results of the paper will be summarized in Section 4.

## 2 METHODS

### 2.1 Full data world/population level

The main focus of survival modeling is on analyzing the effects of a set of covariates $x\in R^{d}$ on a survival time $T\in R^{+}$. We assume that the vector **x**=(X_1_,…,X_d_)^⊤^ has expectation **μ**, covariance matrix **Σ**, and correlation matrix **P**_**X**_. Similarly, we assume that the survival time T has a finite expectation μ_T_ and variance $\sigma_{T}^{2}$. A popular approach to quantify the relationship between T and **x** is the parametric accelerated failure time (AFT) model,^9^ which is based on log‐transformed survival time Y:=log(T) and the model equation

(1) $$Y=\beta_{0}+x^{T}\beta +\epsilon ,$$

where $\beta \in R^{d}$ is a vector of regression coefficients and ϵ is a noise variable. For the derivation of CARS scores, we will consider the special case of lognormally distributed survival times, ie, ϵ is assumed to follow a normal distribution with zero mean and constant variance. Then, the expected squared prediction error $E\;\left({\left(Y-\beta_{0}-x^{T}\beta \right)}^{2}\right)$ is minimized by regression coefficients equal to

(2) $$\beta^{*}=\Sigma^{-1}\Sigma_{XY},$$

where Σ_**X**Y_ is the d‐dimensional vector of covariances between **x** and Y, and the intercept

(3) $$\beta_{0}^{*}=\mu_{Y}-\beta^{T}\mu .$$

Equation (1) is a Gaussian linear regression model. Therefore, in the absence of censoring, a measure of variable importance is the CAR score **θ** 10 defined by

(4) $$\theta =P_{X}^{-1/2}P_{X,Y},$$

where $P_{X}\in R^{d\times d}$ is the correlation matrix of the covariates **x** and $P_{X,Y}\in R^{d\times 1}$ is the vector of correlations between the covariates **x** and the log‐transformed survival time Y=log(T). Analogous to Cox scores, the components of **θ** can be ordered by magnitude to give an importance ranking of the covariates.

The original CAR score for Gaussian linear regression can be interpreted as the correlations between the outcome variable and the decorrelated covariates, which are defined by the orthogonal transformation $z=P_{X}^{-1/2}x$.^10^ Using the best linear unbiased predictor $Y^{⋆}=\beta_{0}^{*}+x^{T}\beta^{*}$ derived from the estimators (2) and (3) and defining $\sigma_{Y}^{2}:=\mathrm{Var}(Y)$, the total variance of Y can be decomposed as follows:

(5) $$\overset{\text{Total variance}}{\overbrace{\mathrm{Var}(Y)}}=\overset{\text{Explained variance}}{\overbrace{\mathrm{Var}(Y^{⋆})}}+\overset{\text{Unexplained variance}}{\overbrace{\mathrm{Var}(Y-Y^{⋆})}}$$

(6) $$\;=\sigma_{Y}^{2}P_{Y,Y^{*}}^{2}+\sigma_{Y}^{2}\left(1-P_{Y,Y^{*}}^{2}\right),$$

where

(7) $$P_{Y,Y^{*}}^{2}=P_{X,Y}^{⊤}P_{X}^{-1}P_{X,Y}=\theta^{T}\theta$$

is the squared correlation between Y and Y ^∗^. From Equations (5) to (7), it follows that the CAR score is the central quantity to assess which variables contribute to the explained variance, or equivalently reduce the unexplained variance. Importantly, $P_{X}^{-1/2}x$ is not the Mahalanobis transform but another form of decorrelation that has the advantageous feature of maximizing the correlation between the decorrelated covariates and the standardized original covariates.^11^ In contrast, the Mahalanobis transform maximizes the cross‐covariance between the decorrelated covariates and the original covariates. Zuber et al^6^, ^10^ demonstrated that the estimated CAR scores result in improved variable rankings and higher predictive performance when compared to other variable selection and modeling techniques, such as lasso or boosting.

Similar to the Elastic Net,^12^ variable selection using the CAR score exhibits the grouping property,^10^ which ensures that highly correlated nonantagonistic variables receive similar CAR scores and are thus close. This property is often desirable from an explanatory modeling perspective.^12^ The grouping property also implies that CAR scores typically do not select a “prototype” out of a group of highly correlated variables, where prototype variable selection may be potentially useful for prediction modeling in some scenarios. Another favorable property of CAR scores is that antagonistic positively correlated variables tend to be bottom ranked and that null predictors (showing no correlation with the response) will be identified (ie, bottom ranked) with probability 1 as long as the employed estimator is consistent. For details on the properties of CAR scores, see the work of Zuber and Strimmer.^10^

Although these estimated scores work well for regression models with a continuous outcome, they were not able to deal with right‐censored data so far. We will therefore develop the CARS score that extends traditional estimators of CAR scores to survival modeling.

### 2.2 Observed data world/sample level

In the observed data world, one often has to deal with right‐censoring, ie, one is no longer able to observe the uncensored survival times of all observations but only the minimum of the true survival time T and a censoring time $C\in R^{+}$. The observed variable of interest is then defined by $\tilde{Y}=\log(\tilde{T})$, where $\tilde{T}:=\min(T,C)$. Additionally, we introduce a status indicator Δ: = I(T ≤ C ), ie, Δ = 1 if the event is observed and Δ = 0 if censoring has occurred. We will further assume that T and C are independent random variables conditional on the covariates **x** (“noninformative censoring”). At the sample level, the empirical CAR score is defined by

(8) $$\hat{\theta }=R_{\text{shrink}}^{-1/2}\;R_{X,Y},$$

where $R_{\text{shrink}}\in R^{d\times d}$ is a shrinkage estimator of the correlation matrix **P**_**X**_ and $R_{X,Y}\in R^{d\times 1}$ is an estimator of the vector of correlations **P**_**X**,Y_.^10^ The definition of **R**_shrink_ will be provided below. In contrast to uncensored Gaussian regression, the standard estimation of **P**_**X**,Y_ using the sample correlations between **x** and $\tilde{Y}$ is no longer appropriate, as this would result in biased estimators in the presence of right‐censoring. To overcome this problem, we suggest adjusting the sample correlations by IPC weighting,^7^ which will result in a consistent estimator of **P**_**X**,Y_.

Definition of IPC weights for right‐censored data: Let ${\tilde{T}}_{1},\text{…},{\tilde{T}}_{n}$ be the observed values of $\tilde{T}$ and C_1_,…,C_n_ be the underlying censoring times in a sample of independent and identically distributed observations of size n. Following the work of Van der Laan and Robins,^7^ we define the IPC weight of the ith observation by

(9) $$w_{i}:=\frac{\Delta_{i}}{{Ĝ}_{n}(\log({\tilde{T}}_{i}))},\;i\in \{1,\text{…},n\},$$

where Δ_i_, i = 1,…,n, are the sample values of Δ and ${Ĝ}_{n}(\cdot )$ is an estimate of the survival function G(·) of the logarithmic censoring process, ie, the probability

(10) G(y)=P(log(C)>y).

By definition, censored observations (Δ_i_ = 0) result in zero IPC weights. In line with the work of Van der Laan and Robins,^7^ we further assume that G(·) > ν > 0, where ν is a small positive real number (this assumption will become important in the consistency proof in Supplementary Appendix A1). To compute ${Ĝ}_{n}(\cdot )$, we apply the Kaplan‐Meier estimator to the observed logarithmic survival times ${\tilde{T}}_{1},\text{…},{\tilde{T}}_{n}$, using the event indicators 1 − Δ_i_, i = 1,…,n.

The definition of the IPC weights in (9) can be extended by allowing the censoring survival function G to conditionally depend on the covariates. For this, it needs to be assumed that ${Ĝ}_{n}(\log(\tilde{T})|x$) is a consistent estimator of the true conditional censoring distribution $G(\log(\tilde{T})|x)$. In practice, ${Ĝ}_{n}(\log(\tilde{T})|x)$ is usually obtained by fitting a multivariable time‐to‐event model to the observed survival times with event indicator 1 − Δ. If the latter model is specified correctly, the following results on the consistency of $\hat{\theta }$ will also hold if G depends on **x**. For details, see Supplementary Appendix A.

Estimation of the correlation vector **P**_**X**,Y_ using IPC weighting: The estimation of **P**_**X**,Y_ comprises the following steps.

1. Estimate the expectations of the covariates X_1_,…,X_d_ by their empirical means ${\bar{X}}_{j}=\sum_{i=1}^{n}X_{i\;j}/n$, j = 1,…,d, where X_i j_ denotes the sample value of the jth covariate in observation i. Similarly, estimate the variances of X_1_,…,X_d_ by their sample variances $S_{j}^{2}=\sum_{i=1}^{n}{\left(X_{i\;j}-{\bar{X}}_{j}\right)}^{2}/(n-1)$, j = 1,…,d.
2. Estimate the expectation of Y by the weighted mean
  (11) $${\bar{Y}}_{w}=\frac{1}{n}\overset{n}{\underset{i=1}{\sum }}w_{i}\log({\tilde{T}}_{i})\;,$$
  where w_i_, i = 1,…,n, are the IPC weights defined in Equation (9). Similarly, estimate the variance of Y by
  (12) $$S_{Y;w}^{2}=\frac{1}{n}\overset{n}{\underset{i=1}{\sum }}w_{i}{\left(\log({\tilde{T}}_{i})-{\bar{Y}}_{w}\right)}^{2}.$$
3. The covariance of X_j_ and Y is again estimated using IPC weighting, ie,
  (13) $$S_{X_{j},Y;w}=\frac{1}{n}\overset{n}{\underset{i=1}{\sum }}w_{i}(X_{i\;j}-{\bar{X}}_{j})\left(\log({\tilde{T}}_{i})-{\bar{Y}}_{w}\right)\;,\;j=1,\text{…},d.$$
4. The final step is to compute the empirical correlation vector **R**_**X**,Y_ by combining the estimators defined in Steps 1 to 3 above, as follows:
  (14) $$R_{X,Y}={\left(\frac{S_{X_{j},Y;w}}{\sqrt{S_{X_{j}}^{2}}\sqrt{S_{Y;w}^{2}}}\right)}_{j=1,\text{…},d}.$$

Estimation of the correlation matrix $P_{X}^{-1/2}$: Since the data values of the covariates are not affected by censoring, the usual sample variance‐covariance estimators could be applied to obtain an estimate of $P_{X}^{-1/2}$. In the presence of high‐dimensional data, however, these estimators usually break down. For example, the estimation of the d × d correlation matrix **P**_**X**_—or, more precisely, its inverse square root—is challenging when d is much larger than the sample size.^13^, ^14^ We therefore propose to employ a shrinkage correlation estimator,^10^, ^15^ to estimate **P**_**X**_, which is given by

(15) $$R_{\text{shrink}}=\lambda I_{d}+(1-\lambda )R_{X},$$

where $\lambda \in R^{+}$ is a shrinkage parameter, **I**_d_ is the identity matrix of dimension d × d, and **R**_**X**_ is the matrix containing the empirical bivariate sample correlations of the covariates. Following the approach in the work of Schäfer and Strimmer,^15^ we define $\lambda :=\sum_{j\neq k}\hat{\mathrm{Var}}(r_{j,\;k})/\sum_{j\neq k}r_{j,\;k}^{2}$, where r_j, k_ denotes the sample correlation between the jth and the kth covariate. The inverse square root $R_{\text{shrink}}^{-1/2}$ can be computed very efficiently by applying singular value decomposition of the sample correlation matrix. For details, we refer to the works of Zuber and Strimmer^10^ and Kessy et al.^11^ The estimation of **P**_**X**,Y_ described above, combined with the shrinkage estimator **R**_shrink_, defines the CARS score in Equation (8).

Consistency of CARS scores: Next, we give a sketch of the consistency proof for the estimated CARS score $\hat{\theta }=R_{\text{shrink}}^{-1/2}\;R_{X,Y}$. As shown in detail in Supplementary Appendix A.4, $\hat{\theta }$ converges to its population value $\theta =P_{X}^{-1/2}\;P_{X,Y}$ as n→∞, provided that (i) censoring is independent of the survival times conditional on **x** and (ii) the estimator ${Ĝ}_{n}$ is a consistent estimator of the censoring survival function G. More specifically, by embedding the IPC‐weighted expressions given in the estimators (11) to (14) in the framework of unbiased estimating equations,^16^, ^17^ we show that each of the estimators contained in the definition of $\hat{\theta }$ is a consistent estimator of its respective population variance or covariance. As a consequence, $\hat{\theta }$ results in a consistent estimator of the population value **θ**.

Variable selection based on CARS scores: As shown above, CARS scores measure the associations between the decorrelated covariates and the time‐to‐event outcome T. Variable selection can therefore be carried out by ranking the CARS scores according to their absolute values. A set of covariates is selected whose absolute CARS scores exceed the predefined threshold value ϕ. A suitable threshold can, for example, be obtained by cross‐validating the multivariable survival model that incorporates the selected covariates. In this paper, we will use a computationally less expensive strategy and apply the adaptive false discovery rate density approach proposed by Strimmer,^18^ which assumes a two‐component discrete mixture model of “influential” and “noninfluential” covariates. On the basis of this model, a suitable threshold value ϕ can be estimated by a trade‐off between the false‐nondiscovery rate P(“influential”|θ ≤ ϕ) and the false‐discovery rate P(“not‐influential”|θ ≥ ϕ). The associated parameters of the mixture model are estimated by penalized maximum likelihood and a modified semiparametric Grenander estimator.^18^, ^19^ For details and for an overview of the advantages of the approach, we refer to the work of Strimmer.^18^

Note that in the derivation of the CARS estimator $\hat{\theta }$, we implicitly assumed that there are no missing values in the data, as pairwise correlations cannot be computed when there are missing values in at least one of the variables. We note that our estimation approach may easily be combined with state‐of‐the‐art methods for dealing with missing covariate values, for example, with multiple imputation^20^ under the missing‐at‐random assumption. After imputation, the CARS estimation approach may be applied to each imputed data set as described above.

## 3 RESULTS

### 3.1 Design of the simulation study

To analyze the performance of CARS scores, we compared the CARS‐based screening approach to a univariate screening approach using Cox scores.^4^ With the latter approach, a univariate Cox model was fitted for each covariate, and the Cox scores were given by the standardized coefficients of these models. In addition, we fitted multivariable Cox regression models with L_1_‐penalized coefficient estimates.^21^ In these models, a variable was considered to be “included” in the model when its L_1_‐penalized coefficient estimate differed from zero. To ensure a fair comparison with the other methods, no tuning of the regularization parameter was applied in the main simulation study, as this would have required additional test data. Instead, we used the median value of the default L_1_‐norm regularization path computed by the R 22 package glmnet.^21^ Furthermore, we carried out an additional simulation study that investigated the impact of tuning multivariable L_1_‐penalized Cox regression models using 10‐fold cross‐validation (see Section 3.5).

We considered three sample sizes ( $n\in \left\{250,500,1000\right\}$) and three dimensions of the covariate space ( $d\in \left\{500,1000,2000\right\}$). The covariate values were generated from a multivariate normal distribution with zero mean. For the covariance structure of the multivariate normal distribution, a block correlation structure with three equally sized blocks was constructed. In the first block, 50% of the correlations were set to ρ = −0.25, and the other 50% were set to ρ = 0.25. In the second block, 50% of the correlations were set to ρ = 0.5, and the other 50% were set to ρ = −0.5. In the third block, 50% of the correlations were set to ρ = 0.75, and the other 50% were set to ρ = −0.75. The correlation between covariates belonging to different blocks was zero. To satisfy the restrictions of a correlation matrix (eg, positive definiteness), the closest correlation matrix with regard to quadratic element‐wise differences was computed. Further details on the algorithm for the construction of the correlation matrix are given in Supplementary Appendix B.7.

The percentages of influential covariates that were related to the time‐to‐event outcome was varied according to the values of 1%, 5%, and 10%. Two different correlation scenarios were analyzed: in the first scenario, all influential variables were taken from the first block of the correlation matrix (“scenario with low absolute correlations”); in the second scenario, all influential variables were taken from the third block (“scenario with high absolute correlations”). The survival process was assumed to follow a lognormal distribution $T∼\log N(\log\mu_{T},\log\sigma_{T}^{2})$ with expectation $\log\mu_{T}=X\beta$. The coefficients **β** were specified to be equidistant within the interval [−0.9, 1], depending on the number of influential covariates. The variance $\log\sigma_{T}^{2}$ was adjusted such that explained variances of {25%,50%,75%} were achieved on the log scale. In another scenario (Section 3.5), Weibull‐distributed survival times T∼W(Φ=exp(Xβ),φ), with scale Φ and shape φ, were explored. In this scenario, the parameter φ was determined by prior simulations with a large data set of 10^6^ observations to achieve a predefined variance, which was determined by a signal‐to‐noise ratio of 0.5. The Weibull scenario was considered because this distribution guarantees that the assumptions of both the AFT model and the Cox model are satisfied at the same time, so that CARS scores and Cox scores were more directly comparable than in the lognormal scenario. The censoring process was assumed to be lognormally distributed. Its parameters were adjusted such that censoring rates of 0.25 and 0.75 were obtained. To emulate administrative censoring, all values of the survival times that were higher than the 90% quantile of the distribution of T were cut off and set to be censored. The 90% quantile was determined by prior simulations with a large sample size of 10^6^ observations for each scenario and remained fixed during simulations. For each of the scenarios, we carried out 300 independent simulation runs.

To evaluate the performance of the methods, the covariates selected by CARS scores, Cox scores, and L_1_‐penalized Cox regression were compared to the sets of influential variables having a true (nonzero) effect on the survival times. For each threshold of the CARS and Cox scores, these comparisons resulted in the cross‐tabulation of the binary variables “selected vs nonselected” and “influential vs noninfluential.” In the case of L_1_‐penalized Cox regression, variables with coefficients different from zero were defined as selected and all other variables as nonselected. Since we were interested in detecting a small set of influential markers within a sparse modeling framework, we used the area under the precision‐recall curve^23^ to evaluate the cross tables and to measure the performance of the three methods. In addition, we investigated the ability of the methods to rank the variables—from least to most important—by analyzing the rank correlations between the true absolute coefficients and the corresponding estimated absolute CARS or Cox scores.

### 3.2 Scenario with low absolute correlations and low censoring rate

**Figure 1:**
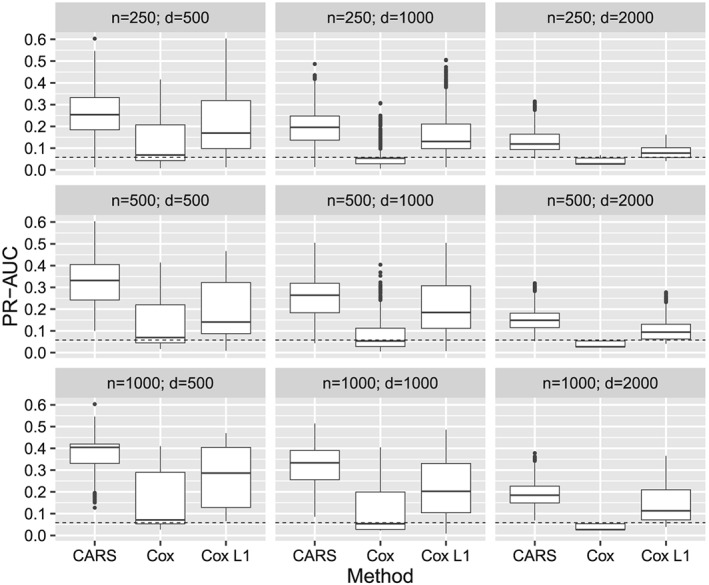
Results of the simulation study. PR‐AUC of CARS, Cox, and Cox L_1_ scores stratified by sample size (n) and number of covariates (d) with low absolute covariate correlations (ρ = ±0.25) and low censoring rate of 25%. Each boxplot summarizes the results of 2700 simulation runs (3 explained variance ratios × 3 signal‐to‐noise ratios × 300 repetitions)

We first present the results of the scenario with low absolute correlations (first block of the correlation matrix, ρ = ±0.25) and 25% censoring rate. After running the algorithm for the construction of the correlation matrix (presented in Supplementary Appendix B.7), all correlations had absolute values that were smaller than 0.3. Figure 1 shows the summary of all simulation results stratified by sample size n and number of covariates d. The median PR‐AUC of the CARS score approach was higher than the respective median PR‐AUC values obtained from Cox scores and L_1_‐penalized Cox regression for all combinations of n and d. In addition, Figure B1 in Supplementary Appendix B.1 displays the results with respect to the relative number of influential variables (relVar) and the explained variance (expVar). In most cases, CARS scores had a clearly higher median PR‐AUC performance than the other approaches, except for the cases where relVar = 0.01 with expVar = 0.5 or expVar = 0.75 and relVar = 0.05 with expVar = 0.75. In these cases, the median PR‐AUC values of CARS and L_1_‐penalized Cox regression were more similar, but CARS scores still outperformed Cox scores. Higher signal‐to‐noise ratios increased the performance of CARS scores on average. The PR‐AUC of CARS scores had a larger variability compared to the Cox‐based approaches, where L_1_‐penalized Cox scores showed a larger variability in PR‐AUC than Cox scores. Further CARS scores ranked influential variables better in the median than Cox scores, as shown in Figure B2 in the Supplementary Appendix. An overall nonstratified summary is available in Supplementary Appendix B.1 (Figure B3).

### 3.3 Scenario with high absolute correlations and low censoring rate

**Figure 2:**
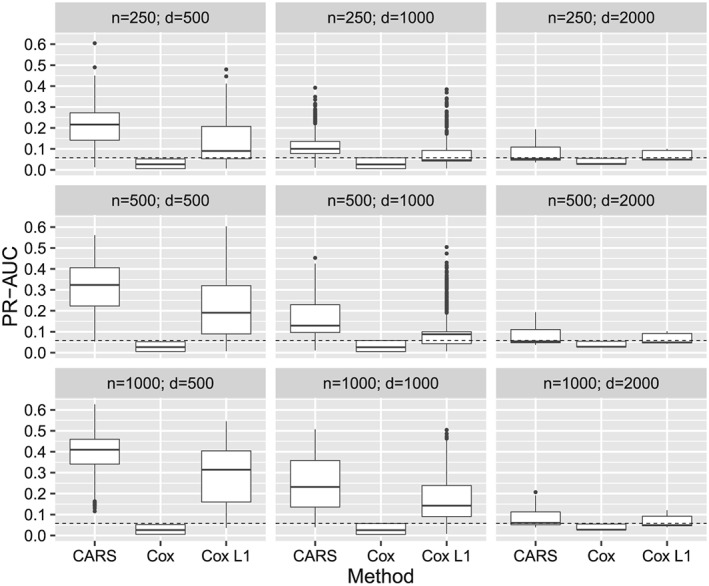
Results of the simulation study. PR‐AUC of CARS, Cox, and Cox L_1_ scores stratified by sample size (n) and number of covariates (d) with high absolute covariate correlations ρ = ±0.75 and censoring rate of 25%. Each boxplot shows the results of 2700 simulation runs (3 explained variance ratios × 3 signal‐to‐noise ratios × 300 repetitions)

Figure 2 presents the results of the scenario with high absolute correlations (third block of the correlation matrix, *ρ* = ±0.75) and with a censoring rate of 25%. The median PR‐AUC of the CARS scores was higher than for both Cox‐based approaches for most combinations of *n* and *d*. Of note, the PR‐AUC performance of Cox scores decreased stronger than the PR‐AUC performance of CARS scores in comparison to the low‐correlation scenario with *ρ* = 0.25. Figure B4 in Supplementary Appendix B.2 displays the results with respect to the number of influential variables and the explained variances. If relVar > 0.05, CARS scores had again higher median PR‐AUC performance than both Cox‐based approaches. The lack of adjustment for between‐covariate correlations obviously degraded the performance of Cox scores. A similar effect was seen in the rank correlations (Figure B5 in the Supplementary Appendix); in particular, the gap between median rank correlations of CARS and Cox scores became somewhat larger compared to the low‐correlation scenario (Figure B2 in the Supplementary Appendix). Figure B6 shows an overall nonstratified summary of the simulation results (Supplementary Appendix B.2).

### 3.4 Scenario with high absolute correlations and high censoring rate

**Figure 3:**
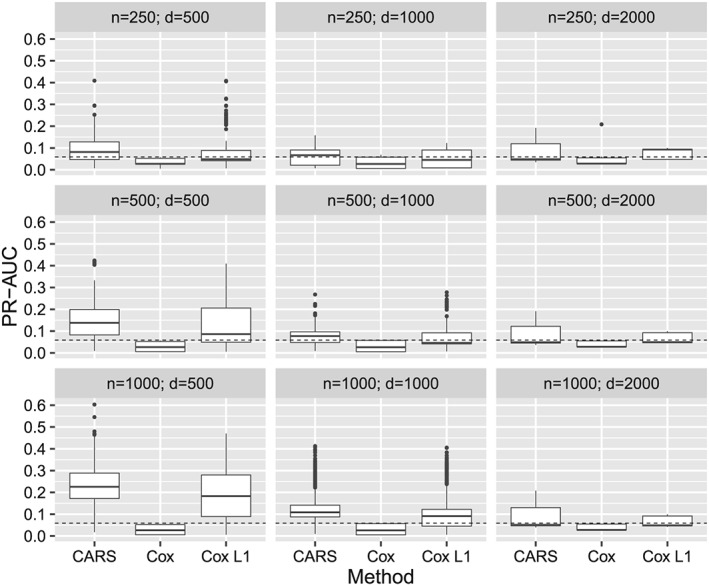
Results of the simulation study. PR‐AUC of all methods stratified by sample size (n) and number of covariates (d) with high absolute covariate correlations of ρ = ±0.75 and censoring rate of 75%. Each boxplot shows the results of 2700 simulation runs (3 explained variance ratios × 3 signal‐to‐noise ratios × 300 repetitions)

Next, we analyzed the results of the scenario with high absolute correlations (third block of the correlation matrix, *ρ* = ±0.75) and with a high censoring rate of 75%. This case is particularly challenging, as approximately 75% of the IPC weights became zero, implying that CARS scores were essentially computed from only 25% of the observations. Consider, for example, the cases *n* < =*d* in Figure 3, where the PR‐AUC performance of all methods was nearly random. If the sample size increased above the number of covariates (ie, *n* > *d*), the median PR‐AUC performance of CARS scores became better than the respective performance of the Cox‐based approaches. Increasing the number of influential covariates from relVaR = 0.01 to relVar = 0.1 improved the PR‐AUC performance of the CARS score approach. Particularly in the cases with relVar = 0.1 and explained variance expVar > =0.5, CARS scores achieved better results in the median than the Cox‐based approaches (see Figure B7 in Supplementary Appendix B.3). Regarding the rank correlations, the CARS score approach behaved better than the Cox score approach (Figure B8 in Supplementary Appendix B.3), although the differences between the approaches were less pronounced than in the previous scenario with high correlations of *ρ* = ±0.75 and low censoring rate of 25%. As in the previous scenarios, Cox scores showed, by far, the worst performance among the three methods on average. Figure B9 shows an overall nonstratified summary of the simulation results (Supplementary Appendix B.3).

### 3.5 Scenario with Weibull‐distributed survival times and low absolute correlations

Here, we present the results of the scenario with low absolute correlations (first block of the correlation matrix, *ρ* = ±0.25) and with 25% censoring rate. In contrast to Section 3.2, the survival times were generated from a Weibull distribution with a logarithmic link function, and the signal‐to‐noise ratio was fixed to 0.5. Moreover, *L*_1_‐penalized Cox regression was additionally tuned by cross‐validation. Figure B10 in Supplementary Appendix B.3 shows the summary of all simulation results. The performance of CARS scores relative to Cox scores and untuned *L*_1_‐penalized Cox regression was essentially comparable to its respective performance in the lognormal scenario, with the CARS score approach performing best in a number of cases. For example, in the cases with *n* = 250 and *d* = 2000, the median CARS PR‐AUC performance was distinctly better than the respective performance of the other methods. Still, the performance of untuned *L*_1_‐penalized Cox regression notably improved (possibly due to the true data‐generating Weibull model being a Cox regression model), with *L*_1_‐penalized Cox regression tending to perform even better in the median—albeit showing a larger variance—than CARS scores when the number of observations increased (*n* = 1000). A comparison of untuned and tuned *L*_1_‐penalized Cox regression shows that the tuned models performed better than the untuned models in the large‐sample‐size scenarios. Of note, the CARS scores outperformed both the tuned and untuned *L*_1_‐penalized Cox models in the higher‐dimensional scenarios (*n* ≤ 500, *d* = 2000).

Figure B11 in the Supplementary Appendix displays the results with respect to the relative number of influential variables (relVar) and the explained variance (expVar). All methods degraded in performance when the number of equicorrelated influential variables (and, thus, multicollinearity) increased, especially the Cox scores. Furthermore, *L*_1_‐penalized Cox regression showed an increased variance, whereas the behavior of the CARS scores was more robust. Again, CARS scores ranked influential variables better in the median than Cox scores, as shown in Figure B12 in the Supplementary Appendix. An overall nonstratified summary is available in Supplementary Appendix B.3 (Figure B13), confirming that tuned *L*_1_‐penalized regression performed slightly better with respect to PR‐AUC than CARS scores in the median but showed a larger variance.

### 3.6 Scenario with Weibull‐distributed survival times and high absolute correlations

Figure B14 in Supplementary Appendix B.3 presents the results of the scenario with high absolute correlations (third block of the correlation matrix, *ρ* = ±0.75) and with a censoring rate of 25%. Overall, the conclusion is similar as in the scenario with lognormal distribution (Section 3.3). The median PR‐AUC of the CARS score approach was throughout higher than the respective median PR‐AUC values of the two Cox‐based approaches for all combinations of *n* and *d*. The PR‐AUC performance of the Cox approaches decreased in comparison to the low‐correlation *ρ* = 0.25 scenario. In contrast to the latter scenario, the untuned *L*_1_‐penalized models performed better throughout than the respective tuned models. Figure B15 in the Supplementary Appendix displays the results with respect to the number of influential variables and the explained variances. For relVar > =0.05, CARS scores had a distinctly higher median PR‐AUC performance than all other methods. Obviously, the lack of adjustment for between‐covariate correlations again degraded the performance of Cox scores. A similar effect was seen in the rank correlations (Figure B16 in the Supplementary Appendix). Figure B14 shows an overall nonstratified summary of the simulation results (Supplementary Appendix B.3).

### 3.7 Runtime in the low‐correlation, low‐censoring scenario

The applicability of CARS scores to high‐dimensional genomic data is determined not only by predictive performance but also by the computational efficiency of CARS score estimation. To analyze the latter, we additionally recorded the runtimes of CARS score, Cox score, and *L*_1_‐penalized Cox estimation without threshold models in the baseline scenario with low covariate correlations *ρ* = ±0.25 and low censoring rate 0.25. All runtimes were recorded using the *R* statistical software without parallelization on the same computer with an Intel Core i7‐7700 CPU at 4.20 GHz and 16‐GB RAM. CARS scores were computed by using the *R* package *carSurv*. Cox scores were calculated using the *R* packages *survival* and *glmnet*. Figure B18 in Supplementary Appendix B.6 shows that in the scenarios with *n* = 500, the average computational time of CARS scores was, in comparison to Cox scores (without the *L*_1_ penalty), about twice as fast to compute. Particularly in the high‐dimensional context with *n* = 250, the average runtimes of the CARS scores were about seven times faster compared to the average Cox score runtimes. In the scenario with *n* = 1000 and *d* = 500, CARS and Cox approaches yielded comparable runtimes, and in the scenarios with *n* = 1000 and *d* > 500, the Cox approaches performed faster.

### 3.8 Application to the Swedish Watchful Waiting Cohort

To investigate the properties of the proposed screening method in a real‐world setting, we applied the CARS score approach to the Swedish Watchful Waiting Cohort data.^2^ The publicly available data consist of 281 patients and 6157 variables that did not contain any missing values. Apart from the clinical covariates (such as patient age, Gleason score, and year of diagnosis), an array of 6100 gene expression profiles (6 K DASL) was designed by using four complementary DNA (cDNA)‐mediated annealing, selection, ligation, and extension (DASL) assay panels (DAPs).^24^, ^25^ Further details of this procedure are available at GeneExpression Omnibus (GEO: http://www.ncbi.nlm.nih.gov/geo/) with platform accession number *GPL5474*. The data are also available at the GEO website with accession number *GSE16560*.

The study population included men who died from prostate cancer during follow‐up or survived at least 10 years after their diagnosis. The sample size was further restricted to men with high‐density tumor regions and who did not receive any type of androgen deprivation. The event of interest was death of prostate cancer; 26.69% of the patients were censored. The median observed time was 100 months (range = [6,274] months). The median age was 74 years (range = [51,91] years), the median Gleason score was seven (range = [2,10]), and 58.72% of the patients had lethal diagnosis. The 2.5% and 97.5% quantiles of the Pearson correlations between the gene expressions were $\left[-0.2634,0.2865\right]$, and the maximum absolute correlation was 0.9861. We applied CARS scores to screen for genes that influenced time to death from prostate cancer and evaluated their performance in comparison to Cox scores.

As the true effects of the genetic markers were unknown, it was not possible to analyze CARS and Cox scores by using the PR‐AUC and rank correlation techniques considered in the previous subsections. Instead, we evaluated the scores by comparing their 10‐times‐repeated 10‐fold cross‐validated predictive performance. The latter was measured by the time‐dependent PR‐AUC,^26^ which is an extension of PR‐AUC to censored data.^7^ The time‐dependent PR‐AUC can be interpreted as the average positive predictive value over time. In addition, we computed a time‐independent summary performance measure by weighting and integrating PR‐AUC over time. In each of the 10 × 10 training folds, CARS and Cox scores were estimated. Each set of risk score values was split into influential and noninfluential genetic markers with a predefined *q* value cutoff threshold *α*_1_ ∈ {0.01,0.05,0.1}. The cutoff threshold was compared to the *q* values given by the method in the work of Strimmer^18^ described in Section 2. For the Cox scores, we used the same threshold procedure as for the CARS scores. All genetic markers with lower *q* values than the specified threshold were selected and incorporated into a multivariable Cox regression model that also included a clinical baseline formula with the variables age, Gleason score, and extremity diagnosis (patient group lethal or indolent).^2^ The performance of a random classifier corresponds to the time‐integrated event rate, which was calculated as *P*(*T* < *t*_0_) averaged over all available time points *t*_0_ within one‐fold. The average of the time‐integrated event rates was computed across the 10 × 10 cross‐validation folds.

**Figure 4:**
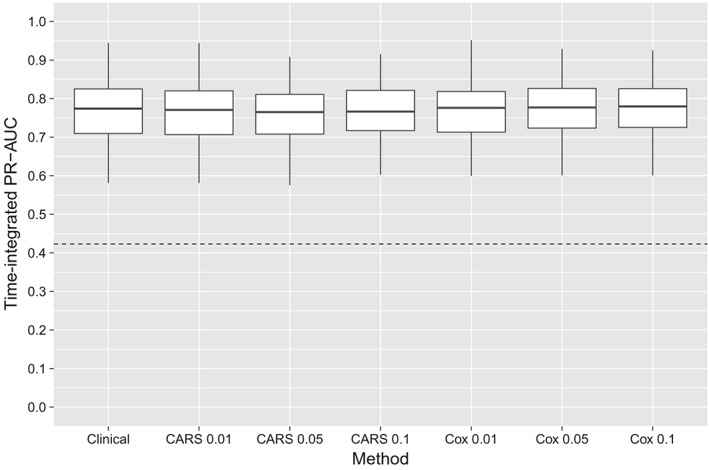
Analysis of the Swedish Watchful Waiting Cohort. The boxplots show the integrated PR‐AUCs of Cox models, as obtained from 10‐times‐repeated 10‐fold cross‐validation. In addition to the clinical baseline formula used in the work of Sboner et al,^2^ the models contained genetic markers that were selected by CARS and Cox scores. The values {0.01,0.05,0.1} represent different q value thresholds. The dashed line denotes the average time‐integrated event rate and corresponds to the performance of a random classifier

Time‐integrated PR‐AUCs for each fold are shown in Figure 4. All methods had higher integrated PR‐AUC values than a random classifier across all cross‐validation folds. Genetic marker selection based on CARS scores resulted in a similar predictive performance compared to genetic marker selection by Cox scores. Both approaches were fairly robust against the choice of the threshold *α*_1_. According to Figure 4, there appears to be no predictive benefit when genetic markers are added to the clinical baseline formula, with CARS scores and Cox scores producing consistent results. This agrees with the findings in the original publication.^2^

The complete data analysis with univariate CARS score screening resulted in 0, 3, and 10 identified genetic markers at the *q* value thresholds *α*_1_ = {0.01,0.05,0.1}, respectively (see Table C1 in the Supplementary Appendix). Genetic marker selection by Cox scores yielded 1, 1, and 2 genetic markers at the same thresholds. Some of the selected genes by CARS scores with *α*_1_ = 0.1 match previous results from the literature: according to the NCBI database,^27^ the “BIRC5 baculoviral IAP repeat containing 5” is an inhibitor of apoptosis and found in most tumor cells. The gene BMX nonreceptor tyrosine kinase regulates differentiation and tumorigenicity of several types of cancer cells, and another gene (MLLT11, transcription factor 7 cofactor) was expressed in several leukemic cell lines.

### 3.9 Application to breast cancer microarray data

In our second real‐world example, we applied CARS and Cox scores to an invasive breast cancer data set collected by Hatzis et al^8^ and Itoh et al.^28^ Merging both available microarray gene expression data sets in the NCBI database^13^ (GEO accession numbers *GSE25055*, *GSE25065*, and *GSE25066*) resulted in 502 observations and 22 338 variables. The latter consisted of 55 clinical and metadata variables and 22 283 gene expression markers. The gene expression data were collected using *GPL96* [HG‐U133A] Affymetrix Human Genome U133A Arrays. There were two patients with missing values that were excluded from statistical analysis. The outcome was the time to distant relapse‐free survival before surgery (median = 2.716 years, range = [0,7.439] years); 21.91% of the patients had a relapse within the study duration. The 2.5% and 97.5% quantiles of the Pearson correlations between the gene expressions were $\left[-0.2467,0.2725\right]$, and the maximum absolute correlation was 0.9986.

Analogous to the previous subsection, we used 10‐times‐repeated 10‐fold cross‐validation to analyze predictive performance. The clinical baseline model included the covariates age, tumor stage, and an indicator of estrogen receptor (ER) positiveness. The genetic markers were selected by either CARS or Cox scores with different *q* value thresholds *α*_1_ = {0.01,0.05,0.1}. The significant genetic markers were added to the clinical covariates, and a multivariable Cox regression model was fitted. Due to the large number of covariates, Cox regression was regularized with an additional *L*_1_ penalty. The regularization parameter was tuned by internal 10‐fold cross‐validation as implemented in the *R* package *glmnet*.

**Figure 5:**
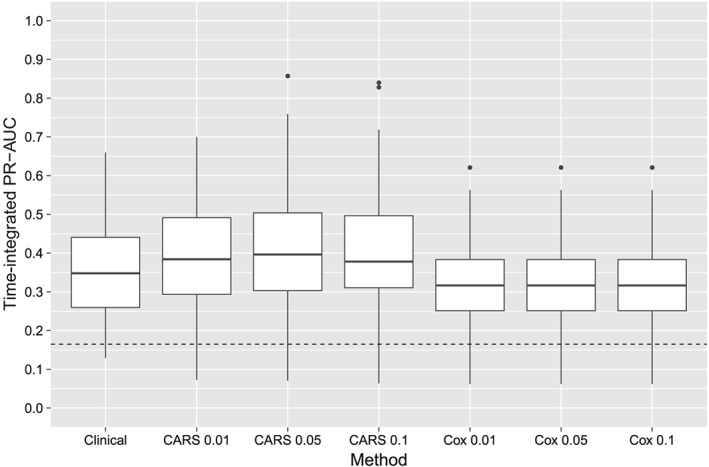
Analysis of the breast cancer data.^8^ The boxplots show the integrated PR‐AUCs of L_1_‐penalized Cox models, as obtained from 10‐times‐repeated 10‐fold cross‐validation. In addition to the clinical baseline formula, the models contained genetic markers that were selected by CARS and Cox scores. The average time‐integrated event rate is displayed as a dashed line, which corresponds to the performance of a random classifier

The predictive performance of Cox regression based on CARS and Cox scores is shown in Figure 5. It is seen that CARS scores performed better than Cox scores for all levels of *α*_1_. For example, when using *α*_1_ = 0.01 as the significance threshold, 22 out of the 22 283 genetic markers were selected by the CARS‐based procedure. Genetic marker selection based on Cox scores identified zero genetic markers at *α*_1_ = 0.01 and failed to include influential genetic markers, which degraded predictive performance. In contrast to the Swedish Watchful Waiting Cohort data, there were notable improvements in predictive performance when the genetic markers were added to the clinical model. All identified genes are presented in the Supplementary Appendix (Table C2).

In order to annotate the 22 genes indicated by the CARS score as highly associated with survival at a *q* value level of *α*_1_ < 0.01, we conducted a gene set enrichment analysis based on gene ontology (GO)^29^ terms as implemented in the Bioconductor^30^ package *topGO*.^31^ The GO framework provides a set of structured vocabularies for specific biological domains that can be used to describe gene products in any organism. We computed Fisher's test for enrichment of molecular function and report in Supplementary Table C3 the 32 GO terms that were enriched at the *p* value significance level *α*_2_ < 0.05. Among the five GO terms that had attained *α*_2_ < 0.01 in the Fisher enrichment test, we detected both protein‐glycine ligase activity and protein‐glycine ligase activity inhibition. Glycine metabolism has been associated with cancer cell proliferation,^32^ and glycine uptake and catabolism can promote tumourigenesis and malignancy.^33^ The third enriched GO term was Ras guanyl‐nucleotide exchange factor activity. Guanyl‐nucleotide exchange factors are proteins that activate GTPases, which are enzymes binding and hydrolizing guanosine triphosphate. Ras is one of the key oncogenes; although Ras mutations are comparatively rare in breast cancer, the RasGAP (Ras GTPase Activating Proteins) gene RASAL2 functions as a tumor suppressor.^34^ Furthermore, we found enrichment evidence for sodium bicarbonate symporter activity, which enables the transfer of a solute or solutes from one side of a membrane to the other and has a central role in pH regulation. Solid tumor exhibits different pH profiles compared to normal tissues, which points at a metabolic shift toward acid‐producing pathways, reflecting both oncogenic signaling and the development of hypoxia.^35^ The sodium bicarbonate cotransporter NBCn1 is the predominant mechanism of acid extrusion in primary breast carcinomas compared to normal tissues affecting intracellular pH levels.^36^ Finally, we detected evidence of estrogen 16‐alpha‐hydroxylase activity, which is one of the earliest reported biomarkers for breast cancer.^37^

## 4 SUMMARY AND DISCUSSION

With high‐dimensional omics data becoming more readily available in medical research, fast and efficient screening methods are needed for statistical model building and prediction. In this paper, we have developed a framework for the selection of genetic markers in time‐to‐event models. This framework helps improve biomarker discovery especially in high‐dimensional settings with a large number of candidate variables. The proposed CARS score, which evaluates the associations between the decorrelated marker values and the time‐to‐event outcome, is estimated consistently by combining a set of IPC‐weighted variance‐covariance estimates. As shown in Section 2, estimates can be computed efficiently even when the number of candidate markers is large. On the basis of the rankings of the CARS score estimates, genetic markers can be selected for inclusion in a multivariable time‐to‐event model, where selection errors can be controlled by the adaptive false discovery rate density approach.^18^

In the numerical experiments presented in Section 3, CARS scores showed promising results with regard to the identification of influential marker variables. In particular, screening based on CARS scores outperformed the traditional screening methods based on Cox scores and showed a better performance than *L*_1_‐penalized regression in many of the analyzed scenarios (although not performing uniformly better, especially in scenarios where the proportional hazards assumption was satisfied, sample sizes were large, and the shrinkage parameter of *L*_1_ penalization was tuned). With regard to predictive performance, the difference between CARS and Cox scores became largest when marker correlations were high. In these situations, the decorrelation of the markers, which is the key feature of CARS scores, had a particularly strong effect on the predictive performance of the multivariable models. Conversely, screening with Cox scores, which ignores the correlations between markers, could not discriminate between noise and influential variables in low and high covariate correlation settings, thereby degrading predictive performance. Since IPC‐weighted estimators tend to have a high variance when censoring rates are high, we also evaluated the proposed estimators in scenarios with censoring rates as high as 75%. Even in these extreme cases, CARS‐based screening did not result in a systematically worse performance than Cox‐based screening.

It should be emphasized that CARS scores are based on the theoretical framework of parametric AFT models. In future research, it will therefore be necessary to further investigate the properties of the CARS score approach in situations where the conditional survival times do not follow an AFT model. In particular, it should be investigated whether it is possible to extend our framework to semiparametric and/or nonlinear regression and different censoring mechanisms. Furthermore, our simulation study did not contain a comparison of the CARS approach to other penalized regression approaches, such as the Elastic Net^12^ or adaptive lasso,^38^ and the effect of hyperparameter tuning in penalized Cox regression was only partially investigated. The censoring distribution influences the estimation of IPC weights, and their impact on CARS performance (eg, in the case of nonlognormal censoring distributions or misspecification of the conditional censoring model) will need further analysis. Evaluating the effect of different distributional assumptions on the covariates will be another topic for future research.

## 5 SOFTWARE

CARS scores are implemented in *R* 22 and published as add‐on package *carSurv* (Version 1.0.0), which is available from CRAN. Other packages used in this article include *survival* (Version 2.41‐3),^39^ *fdrtool*(Version 1.2.15),^40^ *survAUC* (Version 1.0‐5),^41^ *ggplot2* (Version 2.2.1),^42^ *mvnfast*,^43^ *PRROC* (Version 1.3),^44^ and *glmnet* (Version 2.0‐13).^21^

## Supporting information

SIM_8116‐Supp‐0001‐SupplementMaterial.tex

SIM_8116‐Supp‐0002‐SupplementMaterial.pdf

## References

[1] Howlader N , Noone AM , Krapcho M , et al. Seer Cancer Statistics Review, 1975‐2013. Bethesda, MD: National Cancer Institute; 2016 http://seer.cancer.gov/csr/1975_2013/. Access September 30, 2016. Website based on November 2015 SEER data submission.

[2] Sboner A , Demichelis F , Calza St , et al. Molecular sampling of prostate cancer: a dilemma for predicting disease progression. BMC Med Genom. 2010;3(1):1‐12. 10.1186/1755-8794-3-8 PMC2855514 20233430

[3] Cox DR . Regression models and life‐tables. J Royal Stat Soc Ser B. 1972;34(2):187‐220.

[4] Fan J , Lv J . Sure independence screening for ultrahigh dimensional feature space. J Royal Stat Soc Ser B. 2008;70(5):849‐911. 10.1111/j.1467-9868.2008.00674.x PMC2709408 19603084

[5] Benjamini Y , Hochberg Y . Controlling the false discovery rate: a practical and powerful approach to multiple testing. J Royal Stat Soc Ser B. 1995;57(1):289‐300.

[6] Zuber V , Silva PD , Strimmer K . A novel algorithm for simultaneous SNP selection in high‐dimensional genome‐wide association studies. BMC Bioinform. 2012;13(1):284. 10.1186/1471-2105-13-284 PMC3558454 23113980

[7] Van der Laan MJ , Robins JM . Unified Methods for Censored Longitudinal Data and Causality. New York, NY:Springer Science & Business Media; 2003 *Springer Series in Statistics*.

[8] Hatzis Ch , Pusztai L , Valero V , et al. A genomic predictor of response and survival following taxane‐anthracycline chemotherapy for invasive breast cancer. J Am Med Assoc. 2011;305(18):1873‐1881. 10.1001/jama.2011.593 PMC5638042 21558518

[9] Klein JP , Van Houwelingen HC , Ibrahim JG , et al. Handbook of Survival Analysis. London, UK: Chapman & Hall/CRC Press; 2013.

[10] Zuber V , Strimmer K . High‐dimensional regression and variable selection using CAR scores. Stat Appl Genet Mol Biol. 2011;10(34):2194‐6302.

[11] Kessy A , Lewin A , Strimmer K . Optimal whitening and decorrelation. Am Stat. 2018;72(4):309‐314.

[12] Zou H , Hastie T . Regularization and variable selection via the elastic net. J Royal Stat Soc Ser B. 2005;67:301‐320.

[13] Edgar R , Domrachev M , Lash AE . Gene expression omnibus: NCBI gene expression and hybridization array data repository. Nucleic Acids Res. 2002;30(1):207‐210. 11752295 10.1093/nar/30.1.207 PMC99122

[14] Kolesnikov N , Hastings E , Keays M , et al. Arrayexpress—update simplifying data submissions. Nucleic Acids Res. 2014;43(D1):D1113‐D1116. 25361974 10.1093/nar/gku1057 PMC4383899

[15] Schäfer J , Strimmer K . A shrinkage approach to large‐scale covariance matrix estimation and implications for functional genomics. Stat Appl Genet Mol Biol. 2005;4(1):1‐30. 10.2202/1544-6115.1175 16646851

[16] Huber PJ . The behaviour under maximum likelihood estimates under nonstandard conditions In: Proceedings of the Fifth Berkeley Symposium on Mathematical Statistics and Probability, Volume 1: Statistics. Berkeley, CA:University of California Press; 1967.

[17] Carroll RJ , Ruppert D . Transformation and Weighting in Regression. New York, NY: Chapman & Hall/CRC Press; 1988.

[18] Strimmer K . A unified approach to false discovery rate estimation. BMC Bioinform. 2008;9(1):1‐14. 10.1186/1471-2105-9-303 PMC2475539 18613966

[19] Grenander U . On the theory of mortality measurement. Scand Actuar J. 1956;1956(2):125‐153.

[20] van Buuren S . Flexible Imputation of Missing Data. 2nd ed New York: Chapman & Hall/CRC; 2018.

[21] Simon N , Friedman J , Hastie T , Tibshirani R . Regularization paths for Cox's proportional hazards model via coordinate descent. J Stat Softw. 2011;39(5):1‐13. 10.18637/jss.v039.i05 PMC4824408 27065756

[22] R Core Team . R: a language and environment for statistical computing. R Foundation for Statistical Computing. Vienna, Austria; 2017.

[23] Van Rijsbergen CJ . Information Retrieval. 2nd ed. Newton, MA:Butterworth‐Heinemann; 1979.

[24] Fan JB , Yeakley JM , Bibikova M , et al. A versatile assay for high‐throughput gene expression profiling on universal array matrices. Genome Res. 2004;14(5):878‐885. 15123585 10.1101/gr.2167504 PMC479115

[25] Bibikova M , Talantov D , Chudin E , et al. Quantitative gene expression profiling in formalin‐fixed, paraffin‐embedded tissues using universal bead arrays. Am J Pathol. 2004;165(5):1799‐1807. 15509548 10.1016/S0002-9440(10)63435-9 PMC1618650

[26] Yuan Y , Zhou QM , Li B , et al. A threshold‐free summary index of prediction accuracy for censored time to event data. Statist Med. 2018;37(10):1671‐1681. 10.1002/sim.7606 PMC5895543 29424000

[27] NCBI . Database resources of the national center for biotechnology information. Nucleic Acids Res. 2017;45(D1):D12‐D17. 27899561 10.1093/nar/gkw1071 PMC5210554

[28] Itoh M , Iwamoto T , Matsuoka J , et al. Estrogen receptor (ER) mRNa expression and molecular subtype distribution in ER‐negative/progesterone receptor‐positive breast cancers. Breast Cancer Res Treat. 2014;143(2):403‐409. 24337596 10.1007/s10549-013-2763-z

[29] The Gene Ontology Consortium . Creating the gene ontology resource: design and implementation. Genome Res. 2001;11(8):1425‐1433. 11483584 10.1101/gr.180801 PMC311077

[30] Huber W , Carey VJ , Gentleman R , et al. Orchestrating high‐throughput genomic analysis with Bioconductor. Nat Methods. 2015;12(2):115‐121. 25633503 10.1038/nmeth.3252 PMC4509590

[31] Alexa A , Rahnenfuhrer J , Lengauer T . Improved scoring of functional groups from gene expression data by decorrelating go graph structure. Bioinformatics. 2006;22(13):1600‐1607. 16606683 10.1093/bioinformatics/btl140

[32] Amelio I , Cutruzzola F , Antonov A , Agostini M , Melino G . Serine and glycine metabolism in cancer. Trends Biochem Sci. 2014;39(4):191‐198. 24657017 10.1016/j.tibs.2014.02.004 PMC3989988

[33] Jain M , Nilsson R , Sharma S , et al. Metabolite profiling identifies a key role for glycine in rapid cancer cell proliferation. Science. 2012;336(6084):1040‐1044. 22628656 10.1126/science.1218595 PMC3526189

[34] McLaughlin SK , Olsen SN , Dake B , et al. The rasGAP gene, RASAL2, is a tumor and metastasis suppressor. Cancer Cell. 2013;24(3):365‐378. 24029233 10.1016/j.ccr.2013.08.004 PMC3822334

[35] Gorbatenko A , Olesen CW , Boedtkjer E , Pedersen SF . Regulation and roles of bicarbonate transporters in cancer. Front Physiol. 2014;5:130. 24795638 10.3389/fphys.2014.00130 PMC3997025

[36] Boedtkjer E , Moreira JM , Mele M , et al. Contribution of NA^+^,HCO_3_(^−^)‐cotransport to cellular PH control in human breast cancer: a role for the breast cancer susceptibility locus NBCN1 (SLC4A7). Int J Cancer. 2013;132(6):1288‐1299. 22907202 10.1002/ijc.27782

[37] Bradlow HL , Hershcopf R , Martucci C , Fishman J . 16 alpha‐hydroxylation of estradiol: a possible risk marker for breast cancer. Ann NY Acad Sci. 1986;464:138‐151. 3014947 10.1111/j.1749-6632.1986.tb16001.x

[38] Zou H . The adaptive lasso and its oracle properties. J Am Stat Assoc. 2006;101:1418‐1429.

[39] Therneau TM . A package for survival analysis in S. 2015. R Package Version 2.38.

[40] Klaus B , Strimmer K . fdrtool: estimation of (local) false discovery rates and higher criticism. R Package Version 1.2.15. 2015.

[41] Potapov S , Adler W , Schmid M . survAUC: estimators of prediction accuracy for time‐to‐event data. R Package Version 1.0‐5. 2012.

[42] Wickham H . ggplot2: Elegant Graphics for Data Analysis. New York, NY: Springer; 2009.

[43] Fasiolo M . An introduction to mvnfast. R Package Version 0.1.6. 2016.

[44] Grau J , Grosse I , Keilwagen J . PRROC: computing and visualizing precision‐recall and receiver operating characteristic curves in R. Bioinformatics. 2015;31(15):2595‐2597. 25810428 10.1093/bioinformatics/btv153 PMC4514923

