# Supplementary material for: Correlation‐adjusted regression survival scores for high‐dimensional variable selection
Source: Stat Med. 2019 Feb 22;38(13):2413–27. doi: 10.1002/sim.8116 (PMC6519238; doi:10.1002/sim.8116)
Supplement: Supplementary file 2 — SIM_8116‐Supp‐0002‐SupplementMaterial.pdf [file SIM-38-2413-s002.pdf]

# APPENDIX

## A PROOF OF CONSISTENCY OF CAR SURVIVAL SCORES

In this section we prove that the estimator $\hat{\boldsymbol{\theta}}$ of the CAR survival score defined in Equation (8) of the manuscript is consistent for $\boldsymbol{\theta}$. We first define

$$\bar{Y}_w = \frac{1}{n}\sum_{i=1}^{n} w_i \log(\tilde{T}_i), \tag{A1}$$

$$S^2_{Y;w} = \frac{1}{n}\sum_{i=1}^{n} w_i \left(\log(\tilde{T}_i) - \bar{Y}_w\right)^2, \tag{A2}$$

$$\bar{X}_j = \frac{1}{n}\sum_{i=1}^{n} X_{i,j}, \tag{A3}$$

$$S^2_{X_j} = \frac{1}{n-1}\sum_{i=1}^{n} \left(X_{i,j} - \bar{X}_j\right)^2, \tag{A4}$$

$$S_{X_j,Y;w} = \frac{1}{n}\sum_{i=1}^{n} w_i (X_{ij} - \bar{X}_j)\left(\log(\tilde{T}_i) - \bar{Y}_w\right), \tag{A5}$$

$$\boldsymbol{R}_{\boldsymbol{X},Y} = \left(\frac{S_{X_j,Y;w}}{\sqrt{S^2_{X_j}}\sqrt{S^2_{Y;w}}}\right)_{j=1,\ldots,d}, \tag{A6}$$

where $\tilde{T}_i$ and $X_{ij}$, $i = 1, \ldots, n$, $j = 1, \ldots, d$, refer to the sample variables of the observed response and the covariates, respectively. The proof is partitioned into four parts: First the consistency of the IPC weighted mean $\bar{Y}_w$ is shown (Section A.1). Second the consistency of the weighted sample variance $S^2_{Y;w}$ of the response is shown (Section A.2). Third the consistency of the weighted sample covariance $S_{X_j,Y;w}$ of the response and the covariates is shown (Section A.3). In the last part the consistency of $\hat{\boldsymbol{\theta}}$ is derived by combining all previous parts (Section A.4).

### A.1 Consistency of the IPC weighted mean

To show the consistency of $\bar{Y}_w$ it is sufficient to embed this estimator in the framework of unbiased estimation equations.[1] Generally, the estimating equation

$$\frac{1}{n}\sum_{i=1}^{n} \psi(\tilde{Y}_i, w_i, \Theta) = 0 \tag{A7}$$

for some parameter $\Theta$ (here, $\Theta \equiv \mu_Y = E(Y)$) is unbiased if

$$E\left(\psi(\tilde{Y}_i, w_i, \Theta)\right) = 0 \quad \forall\, i = 1, \ldots, n, \tag{A8}$$

where $\psi$ is a real-valued function of $\tilde{Y}_i$, $w_i$ and $\Theta$. The IPC weights $w_i$ are defined as in Equation (9) of the manuscript, i.e.,

$$w_i = \frac{I(C_i \geq T_i)}{\hat{G}_n(\log(\tilde{T}_i))}, \tag{A9}$$

where $\hat{G}_n$ is the Kaplan-Meier estimator of the survival function of the censoring distribution. Note that the Kaplan-Meier estimator is consistent for the true survival function $G$ and can therefore be replaced by $G$ in asymptotic analysis. (This will be done in the following, see also Carroll et al. (2006).[2]) Under some regularity conditions[1] (e.g. measureability, continuity and uniqueness of solutions) the estimator $\hat{\Theta}$ solving (A7) is consistent for $\Theta$ if Equation (A8) holds.

As stated in Section 2.2 of the manuscript, the above scenario can be extended by allowing the censoring survival function to depend on the covariates. For this it needs to be assumed that $\hat{G}_n(\log(\tilde{T}_i)|\boldsymbol{x}_i)$ is a consistent estimator of the true conditional

censoring distribution $G(\log(\tilde{T}_i)|\boldsymbol{x}_i)$, where $\boldsymbol{x}_i$ is a vector containing the sample variables $X_{i1}, \ldots, X_{id}$. In practice, $\hat{G}_n$ is usually obtained by fitting a multivariable time-to-event model to the survival times with event indicator $1 - \Delta$. Correct specification of this model requires, in particular, the selection of the correct set of variables to include in the model. It should be noted that the covariates to be included in the censoring model do not necessarily have to be the same as the covariates considered for CARS score estimation. In IPC weighting, which bears similarities to propensity score weighting in retrospective epidemiological studies,[3] the covariates for the censoring model are often selected a priori based on context knowledge (e.g., demographic covariates and context-specific clinical risk factors). If an a priori choice is not possible and if the number of candidate variables for the censoring model is large, data-driven variable selection for the censoring model (which is a largely unexplored topic in survival analysis) may be done along similar lines as the ones proposed by Brookhart et al. (2006) and Schneeweiss (2009)[3,4] for propensity score modeling.

From now on assume that $G(\cdot|\boldsymbol{x})$ is known and that $G(\cdot|\boldsymbol{x}) > \nu > 0$ for a small real number $\nu$. Next consider the estimation function

$$\psi(\tilde{Y}_i, w_i, \mu_{\log(T)}) = w_i \log(\tilde{T}_i) - \mu_{\log(T)} \tag{A10}$$

for the weighted mean $\bar{Y}_w$. By definition, one obtains

$$E\left(w_i \log(\tilde{T}_i)\right) = \int \int_{-\infty}^{\infty} \int_{-\infty}^{\infty} \frac{I\left(\log(C_i) \geq \log(T_i)\right)}{G(\log(\tilde{T}_i)|\boldsymbol{x}_i)} \log(\tilde{T}_i) f_{\log(T)|\boldsymbol{x}}(\log(T))$$
$$f_{\log(C)|\boldsymbol{x}}(\log(C))\ f_{\boldsymbol{x}}(\boldsymbol{x})\ d\log(T)\ d\log(C)\ d\boldsymbol{x}\,, \tag{A11}$$

where the first integral is over the covariates $\boldsymbol{x} \in \mathbb{R}^d$, where $f_{\log(T)|\boldsymbol{x}}$ and $f_{\log(C)|\boldsymbol{x}}$ denote the conditional probability density functions of $\log(T)|\boldsymbol{x}$ and $\log(C)|\boldsymbol{x}$, respectively, and where $f_{\boldsymbol{x}}$ is the probability density function of the covariates $\boldsymbol{x}$. The following steps show that (A10) defines an unbiased estimation equation for the parameter $\mu_Y \equiv \mu_{\log(T)}$:

$$E\left(w_i \log(\tilde{T}_i)\right) = \int \int_{-\infty}^{\infty} \frac{1}{G(\log(T_i)|\boldsymbol{x}_i)} \log(T_i) f_{\log(T)|\boldsymbol{x}}(\log(T))$$
$$\int_{\log(T_i)}^{\infty} f_{\log(C)|\boldsymbol{x}}(\log(C))\ d\log(C) f_{\boldsymbol{x}}(\boldsymbol{x})\ d\log(T)\ d\boldsymbol{x} \tag{A12}$$
$$= \int \int_{-\infty}^{\infty} \frac{G(\log(T_i)|\boldsymbol{x}_i)}{G(\log(T_i)|\boldsymbol{x}_i)} \log(T_i) f_{\log(T)|\boldsymbol{x}}(\log(T))\ f_{\boldsymbol{x}}(\boldsymbol{x})\ d\log(T)\ d\boldsymbol{x} \tag{A13}$$
$$= \mu_{\log(T)}\,. \tag{A14}$$

## A.2 Consistency of the IPC weighted variance

To prove the consistency of the IPC weighted variance $S^2_{Y;w}$ for $\sigma^2_Y = \sigma^2_{\log(T)}$, we consider the estimation function

$$\psi\left(\tilde{Y}_i, w_i, \sigma^2_{\log(T)}\right) = w_i \left(\log(\tilde{T}_i) - \mu_{\log(T)}\right)^2 - \sigma^2_{\log(T)}\,, \tag{A15}$$

where, compared to the definition of $S^2_{Y;w}$ in (A2), the weighted mean $\bar{Y}_w$ has been replaced by the true expectation $\mu_{\log(T)}$.

The following steps show that (A15) defines an unbiased estimation equation for the parameter $\sigma^2_Y \equiv \sigma^2_{\log(T)}$:

$$E\left(w_i\left(\log(\tilde{T}_i)-\mu_{\log(T)}\right)^2\right)=\int\int_{-\infty}^{\infty}\frac{1}{G(\log(T_i)|\boldsymbol{x}_i)}\left(\log(T_i)-\mu_{\log(T)}\right)^2 f_{\log(T)|\boldsymbol{x}}\left(\log(T)\right)$$

$$\int_{\log(T_i)}^{\infty} f_{\log(C)|\boldsymbol{x}}\left(\log(C)\right)\; d\log(C)\; f_{\boldsymbol{x}}(\boldsymbol{x})\; d\log(T)\; d\boldsymbol{x} \tag{A16}$$

$$=\int\int_{-\infty}^{\infty}\frac{G(\log(T_i)|\boldsymbol{x}_i)}{G(\log(T_i)|\boldsymbol{x}_i)}\left(\log(T_i)-\mu_{\log(T)}\right)^2 f_{\log(T)|\boldsymbol{x}}\left(\log(T)\right)\; f_{\boldsymbol{x}}(\boldsymbol{x})\; d\log(T)\; d\boldsymbol{x} \tag{A17}$$

$$=\sigma^2_{\log(T)}\,. \tag{A18}$$

As shown, for example, in Carroll et al. (2006),[2] the estimator of $\sigma^2_{\log(T)}$ solving $1/n\sum_i \psi\left(\tilde{Y}_i, w_i, \sigma^2_{\log(T)}\right)=0$ remains a consistent estimator if the true expectation $\mu_{\log(T)}$ in (A15) is replaced by the weighted mean $\bar{Y}_w$ in the definition of $S^2_{Y;w}$. This is because $\bar{Y}_w$ is a consistent estimator of $\mu_{\log(T)}$, as shown above.

## A.3 Consistency of the IPC weighted covariance

Following the same strategy as in the previous Section A.2, we consider the following estimation function for the covariance $\sigma_{X_j,Y}\equiv\sigma_{X_j,\log(T)}$ between covariate $X_j$, $j\in\{1,\ldots,d\}$, and $Y$:

$$\psi\left(\tilde{Y}_i, w_i, \sigma_{X_j,\log(T)}\right)=w_i(X_{ij}-\mu_j)\left(\log(\tilde{T}_i)-\mu_{\log(T)}\right)-\sigma_{X_j,\log(T)}\,, \tag{A19}$$

where, compared to the definition of $S_{X_j,Y;w}$ in (A5), the weighted mean $\bar{Y}_w$ and the mean $\bar{X}_j$ have been replaced by the true expectations $\mu_{\log(T)}$ and $\mu_j$, respectively.

The following steps show that (A19) defines an unbiased estimation equation for the parameter $\sigma_{X_j,\log(T)}$:

$$E\left(w_i(X_{ij}-\mu_j)\left(\log(\tilde{T}_i)-\mu_{\log(T)}\right)\right)=\int\int_{-\infty}^{\infty}\frac{1}{G(\log(T_i)|\boldsymbol{x}_i)}(X_{ij}-\mu_j)\left(\log(T_i)-\mu_{\log(T)}\right) f_{\log(T)|\boldsymbol{x}}\left(\log(T)\right)$$

$$\int_{\log(T_i)}^{\infty} f_{\log(C)|\boldsymbol{x}}\left(\log(C)\right)\; d\log(C)\; f_{\boldsymbol{x}}(\boldsymbol{x})\; d\log(T)\; d\boldsymbol{x} \tag{A20}$$

$$=\int\int_{-\infty}^{\infty}\frac{G(\log(T_i)|\boldsymbol{x}_i)}{G(\log(T_i)|\boldsymbol{x}_i)}(X_{ij}-\mu_j)\left(\log(T_i)-\mu_{\log(T)}\right) f_{\log(T)|\boldsymbol{x}}\left(\log(T)\right) f_{\boldsymbol{x}}(\boldsymbol{x})\; d\log(T)\; d\boldsymbol{x} \tag{A21}$$

$$=\sigma^2_{X_j,\log(T)}\,. \tag{A22}$$

Analogous to the previous subsection, the estimator of $\sigma_{X_j,\log(T)}$ solving $1/n\sum_i \psi\left(\tilde{Y}_i, w_i, \sigma_{X_j,\log(T)}\right)=0$ remains a consistent estimator if the true expectations $\mu_j$ and $\mu_{\log(T)}$ in (A19) are replaced by the mean $\bar{X}_j$ and the weighted mean $\bar{Y}_w$, respectively, in the definition of $S_{X_j,Y;w}$. This is because $\bar{X}_j$ and $\bar{Y}_w$ are consistent estimators of $\mu_j$ and $\mu_{\log(T)}$, respectively.

## A.4 Combination of results

In this subsection all previous consistency proofs for the weighted mean, weighted variance and weighted covariance are combined to prove the consistency of the CARS score estimator $\hat{\boldsymbol{\theta}}=\boldsymbol{R}^{-1/2}_{\text{shrink}}\boldsymbol{R}_{\boldsymbol{X},Y}$. First consider the quantity $\boldsymbol{R}_{\boldsymbol{X},Y}$. Denoting by $\boldsymbol{P}_{\boldsymbol{X},Y}$ the vector of true pairwise correlations between the covariates and the response, it follows from the previous subsections that

$$\lim_{n\to\infty}\boldsymbol{R}_{\boldsymbol{X},Y}=\lim_{n\to\infty}\left(\frac{s_{Y,X_j;w}}{s_{X_j}s_{Y;w}}\right)_{j=1,\ldots,d}=\boldsymbol{P}_{\boldsymbol{X},Y}\,. \tag{A23}$$

Next consider the shrinkage estimator $\boldsymbol{R}_{\text{Shrink}}$ [5] of the correlations between the covariates $\boldsymbol{R}_{\boldsymbol{X}}$, which is defined by

$$\boldsymbol{R}_{\text{Shrink}} = \lambda \boldsymbol{I}_d + (1-\lambda)\boldsymbol{R}_{\boldsymbol{X}}\,, \tag{A24}$$

where the shrinkage parameter $\lambda$ is estimated by

$$\hat{\lambda} = \frac{\sum_{j\neq k} \widehat{Var}(\hat{r}_{j,k})}{\sum_{j\neq k} \hat{r}^2_{j,k}} \tag{A25}$$

and

$$\hat{r}_{j,k} = \frac{\frac{1}{n-1}\sum_{i=1}^{n}\left(x_{i,j}-\bar{x}_j\right)\left(x_{i,k}-\bar{x}_k\right)}{\sqrt{\frac{1}{n-1}\sum_{i=1}^{n}\left(x_{i,j}-\bar{x}_j\right)^2}\sqrt{\frac{1}{n-1}\sum_{i=1}^{n}\left(x_{i,k}-\bar{x}_k\right)^2}} \tag{A26}$$

is the sample correlation between the $j$-th and $k$-th covariate. In the limit, the estimator $\hat{\lambda}$ converges to zero, as

$$\lim_{n\to\infty}\hat{\lambda} = \frac{\lim_{n\to\infty}\sum_{j\neq k}\widehat{Var}(\hat{r}_{j,k})}{\lim_{n\to\infty}\sum_{j\neq k}\hat{r}^2_{j,k}} \tag{A27}$$

$$= \frac{\sum_{j\neq k}\lim_{n\to\infty}\widehat{Var}(\hat{r}_{j,k})}{\sum_{j\neq k}\lim_{n\to\infty}\hat{r}^2_{j,k}} \tag{A28}$$

$$= \frac{\sum_{j\neq k} 0}{\sum_{j\neq k} r^2_{j,k}} = 0\,. \tag{A29}$$

As a consequence, $\boldsymbol{R}_{\text{Shrink}}$ approaches the estimator $\boldsymbol{R}_{\boldsymbol{X}}$ in the limit, which is itself a consistent estimator of the covariates' correlation matrix $\boldsymbol{P}_{\boldsymbol{X}}$. Combining all previous results, and applying the continuous mapping theorem as well as Slutsky's theorem, proves the consistency of the CARS score estimator $\hat{\boldsymbol{\theta}} = \boldsymbol{R}^{-1/2}_{\text{shrink}}\boldsymbol{R}_{\boldsymbol{X},Y}$ for the population level CARS score $\boldsymbol{\theta} = \boldsymbol{P}^{-1/2}_{\boldsymbol{X}}\boldsymbol{P}_{\boldsymbol{X},Y}$.

# B ADDITIONAL RESULTS OBTAINED FROM THE SIMULATION STUDY

## B.1 CARS simulation with low absolute covariate correlations

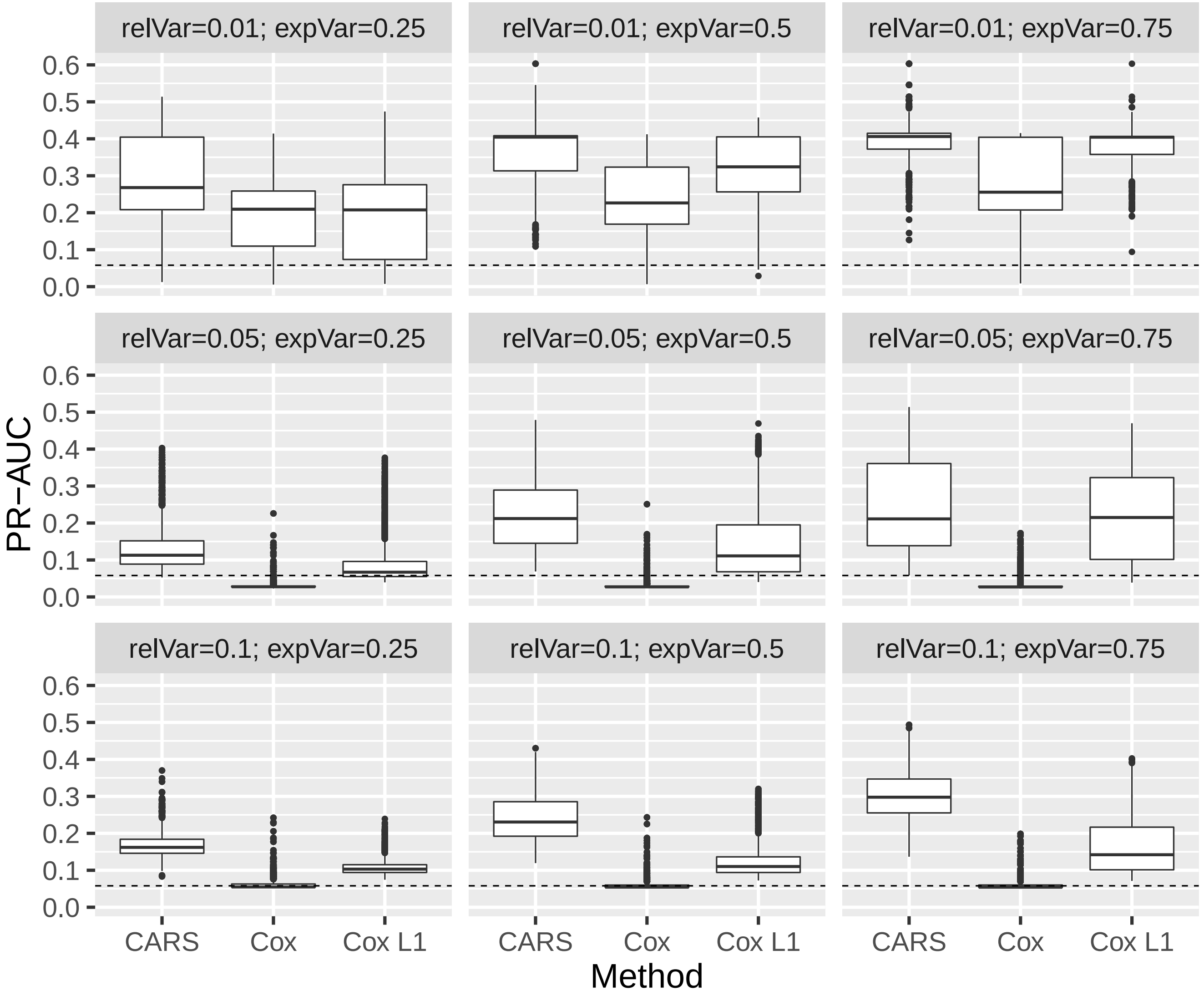


**FIGURE B1** Results of the simulation study. PR-AUC of CARS, Cox and Cox $L_1$ scores stratified by relative number of relevant variables (relVar) and explained variance (expVar) with low absolute covariate correlations ($\rho = \pm 0.25$) and censoring rate of 25%. Each boxplot summarizes the results of 2700 simulation runs (3 sample sizes x 3 number of covariates x 300 repetitions).

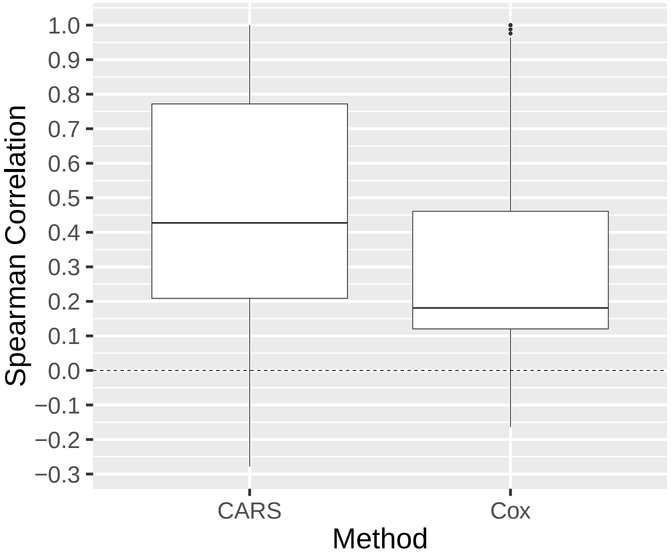


**FIGURE B2** Results of the simulation study, scenario with low absolute correlations ($\rho = \pm 0.25$). The boxplots visualize the rank correlations of the estimated and the true covariate orderings, as obtained from variable selection by CARS and Cox scores. The censoring rate was equal to 25%. Each boxplot shows the results of 24300 simulation runs (3 explained variance ratios x 3 signal to noise ratios x 3 sample sizes x 3 number of covariates x 300 repetitions).

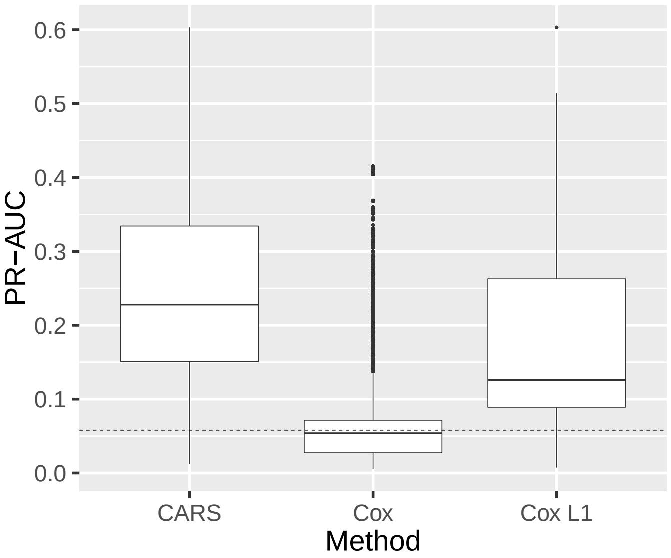


**FIGURE B3** Results of the simulation study, scenario with low absolute correlations ($\rho = \pm 0.25$). The boxplots visualize the PR-AUC values obtained from variable selection by CARS scores, Cox scores and $L_1$-penalized Cox regression. The censoring rate was equal to 25%. The average prevalance of the positive class (computed from all simulations) is displayed by the dashed line. Note that the boxplots contain the PR-AUC values corresponding to all three rates of influential covariates (1%, 5%, 10%). Each boxplot shows the results of 24300 simulation runs (3 explained variance ratios x 3 signal to noise ratios x 3 sample sizes x 3 number of covariates x 300 repetitions).

## B.2 CARS simulation with high absolute covariate correlations

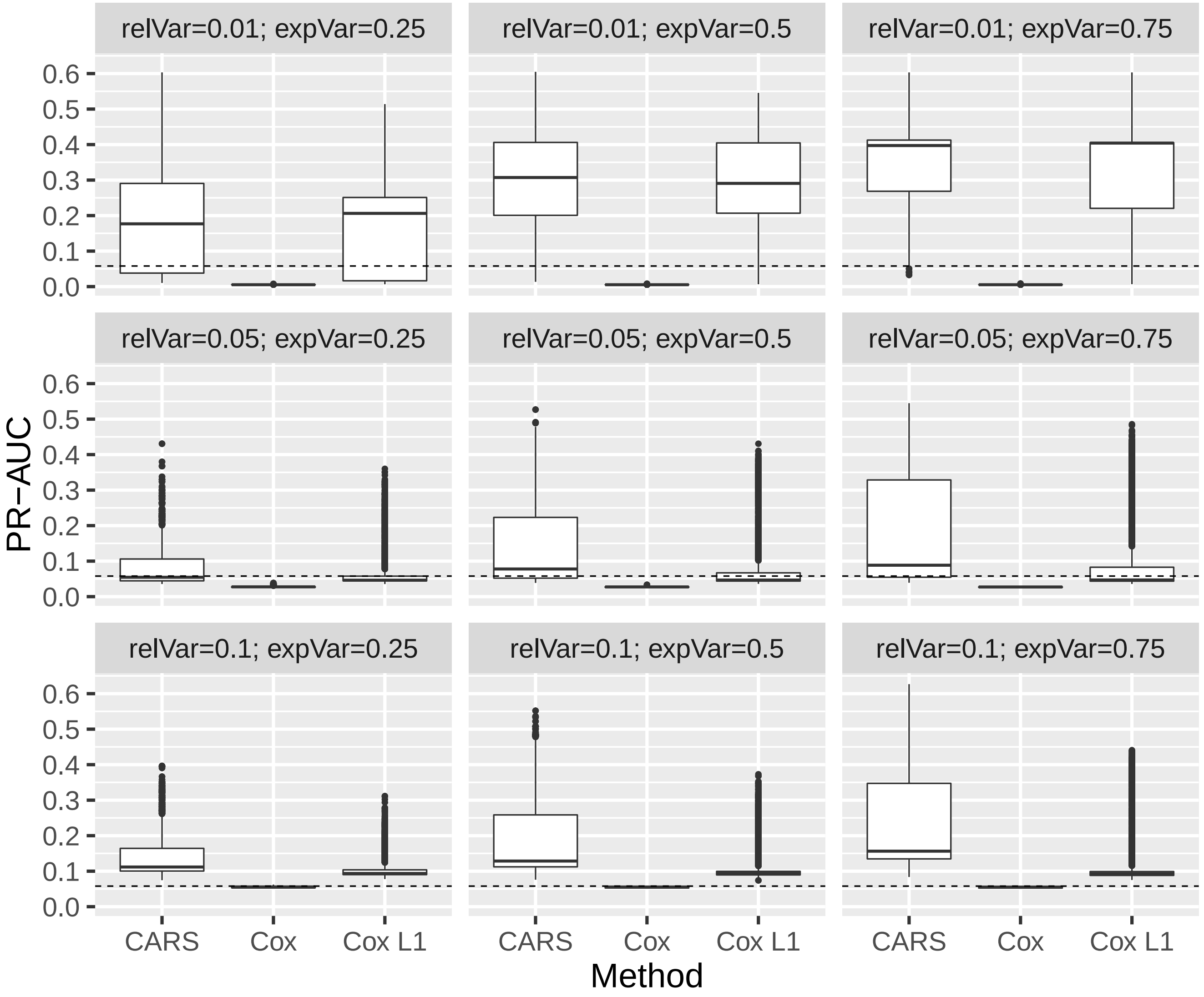


**FIGURE B4** Results of the simulation study. PR-AUC of CARS, Cox and Cox $L_1$ scores stratified by relative number of relevant variables (relVar) and explained variance (expVar) with high absolute covariate correlations $\rho = \pm 0.75$ and censoring rate of 25%. Each boxplot shows the results of 2700 simulation runs (3 sample sizes x 3 number of covariates x 300 repetitions).

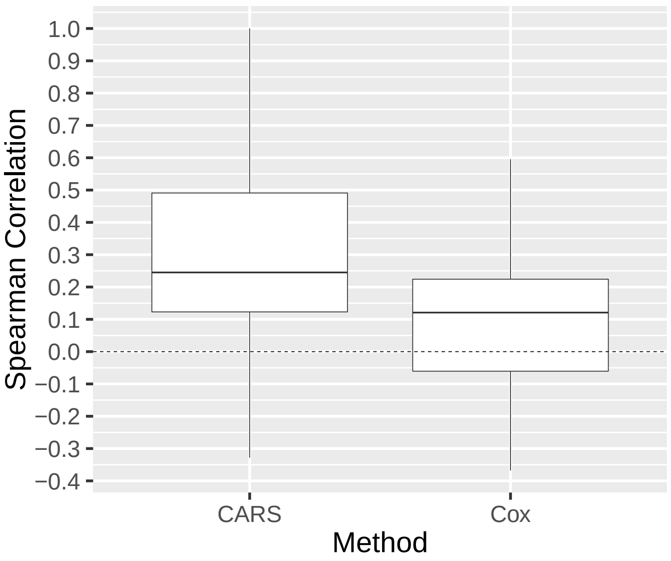


**FIGURE B5** Results of the simulation study, scenario with high absolute correlations ($\rho = \pm 0.75$). The boxplots visualize the rank correlations of the estimated and the true covariate orderings, as obtained from variable selection by CARS and Cox scores. The censoring rate was equal to 25%. Each boxplot shows the results of 24300 simulation runs (3 explained variance ratios x 3 signal to noise ratios x 3 sample sizes x 3 number of covariates x 300 repetitions).

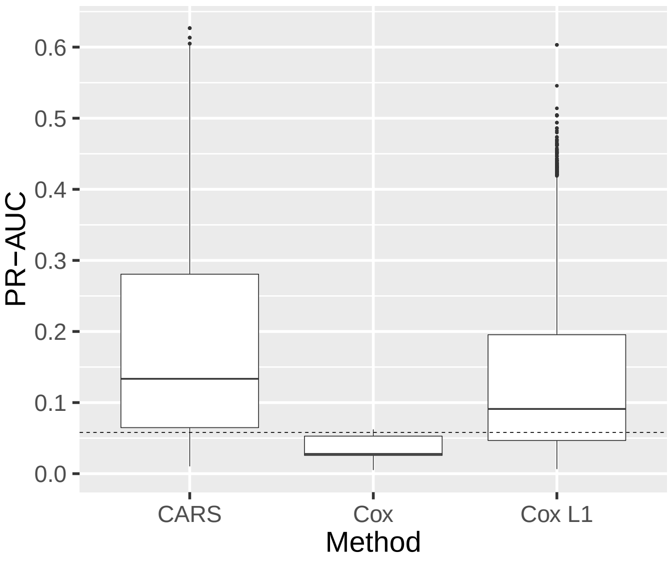


**FIGURE B6** Results of the simulation study, scenario with high absolute correlations ($\rho = \pm 0.75$). The boxplots visualize the PR-AUC values obtained from variable selection by CARS scores, Cox scores and $L_1$-penalized Cox regression. The censoring rate was equal to 25%. The average prevalance of the positive class (computed from all simulations) is displayed by the dashed line. Note that the boxplots contain the PR-AUC values corresponding to all three rates of influential covariates (1%, 5%, 10%). Each boxplot shows the results of 24300 simulation runs (3 explained variance ratios x 3 signal to noise ratios x 3 sample sizes x 3 number of covariates x 300 repetitions). Note that almost all PR-AUC values of the Cox score approach are below the PR-AUC of a random classifier.

### B.3 CARS simulation with high absolute covariate correlations and censoring rate 0.75

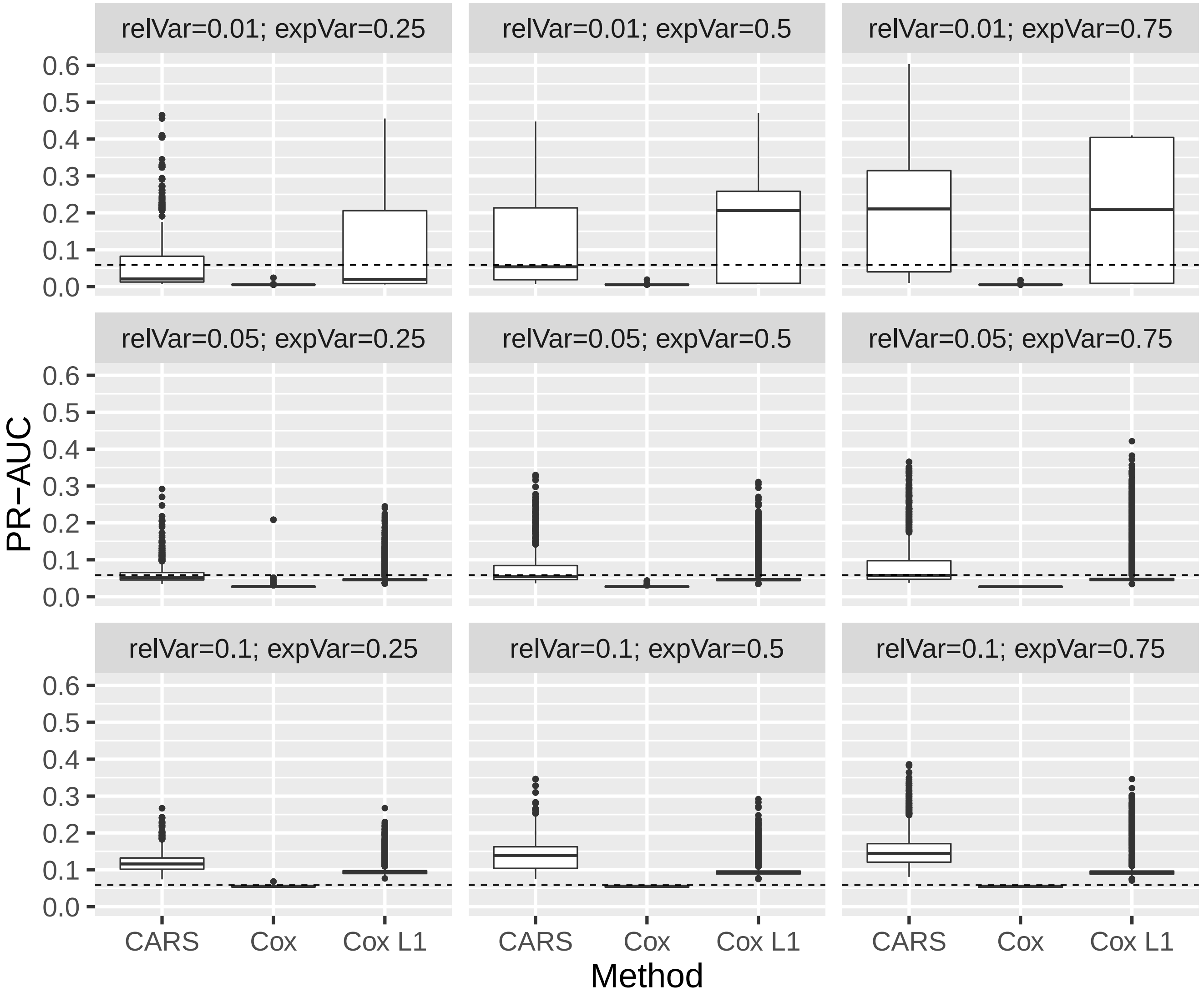


**FIGURE B7** Results of the simulation study. PR-AUC of CARS, Cox and Cox $L_1$ scores stratified by relative number of relevant variables (relVar) and explained variance (expVar) with high absolute covariate correlations ($\rho = \pm 0.75$) and censoring rate of 75%. Each boxplot shows the results of 2700 simulation runs (3 sample sizes x 3 number of covariates x 300 repetitions).

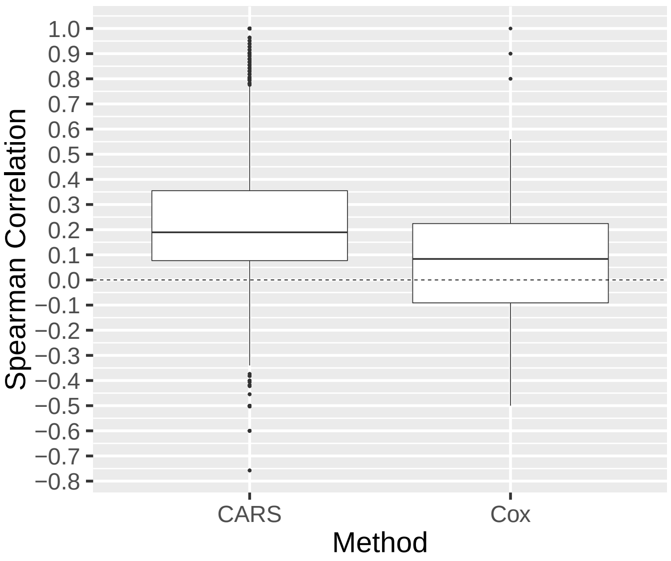


**FIGURE B8** Results of the simulation study, scenario with high absolute correlations ($\rho = \pm 0.75$) and a high censoring rate of 75%. The boxplots visualize the rank correlations of the estimated and the true covariate orderings, as obtained from variable selection by CARS and Cox scores. Each boxplot shows the results of 24300 simulation runs (3 explained variance ratios x 3 signal to noise ratios x 3 sample sizes x 3 number of covariates x 300 repetitions).

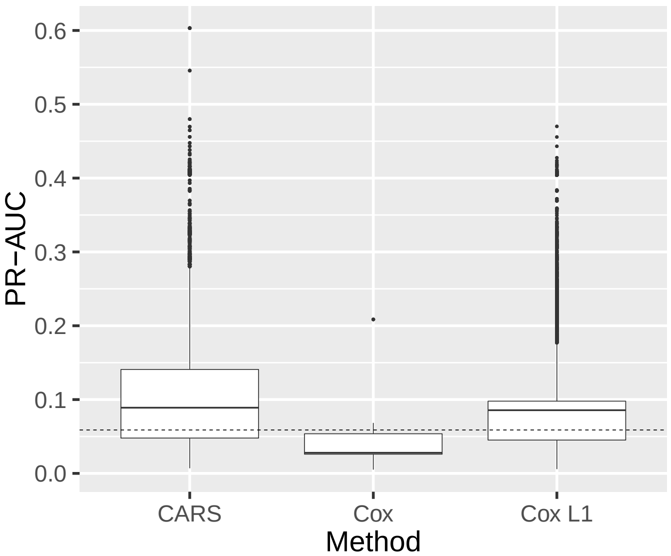


**FIGURE B9** Results of the simulation study, scenario with high absolute correlations ($\rho = \pm 0.75$) and a high censoring rate of 75%. The boxplots visualize the PR-AUC values obtained from variable selection by CARS scores, Cox scores and $L_1$-penalized Cox regression. The average prevalance of the positive class (computed from all simulations) is displayed by the dashed line. Note that the boxplots contain the PR-AUC values corresponding to all three rates of influential covariates (1%, 5%, 10%). Each boxplot shows the results of 24300 simulation runs (3 explained variance ratios x 3 signal to noise ratios x 3 sample sizes x 3 number of covariates x 300 repetitions). Note that almost all PR-AUC values of the Cox score approach are below the PR-AUC of a random classifier.

## B.4 CARS simulation with low absolute covariate correlations and Weibull distribution

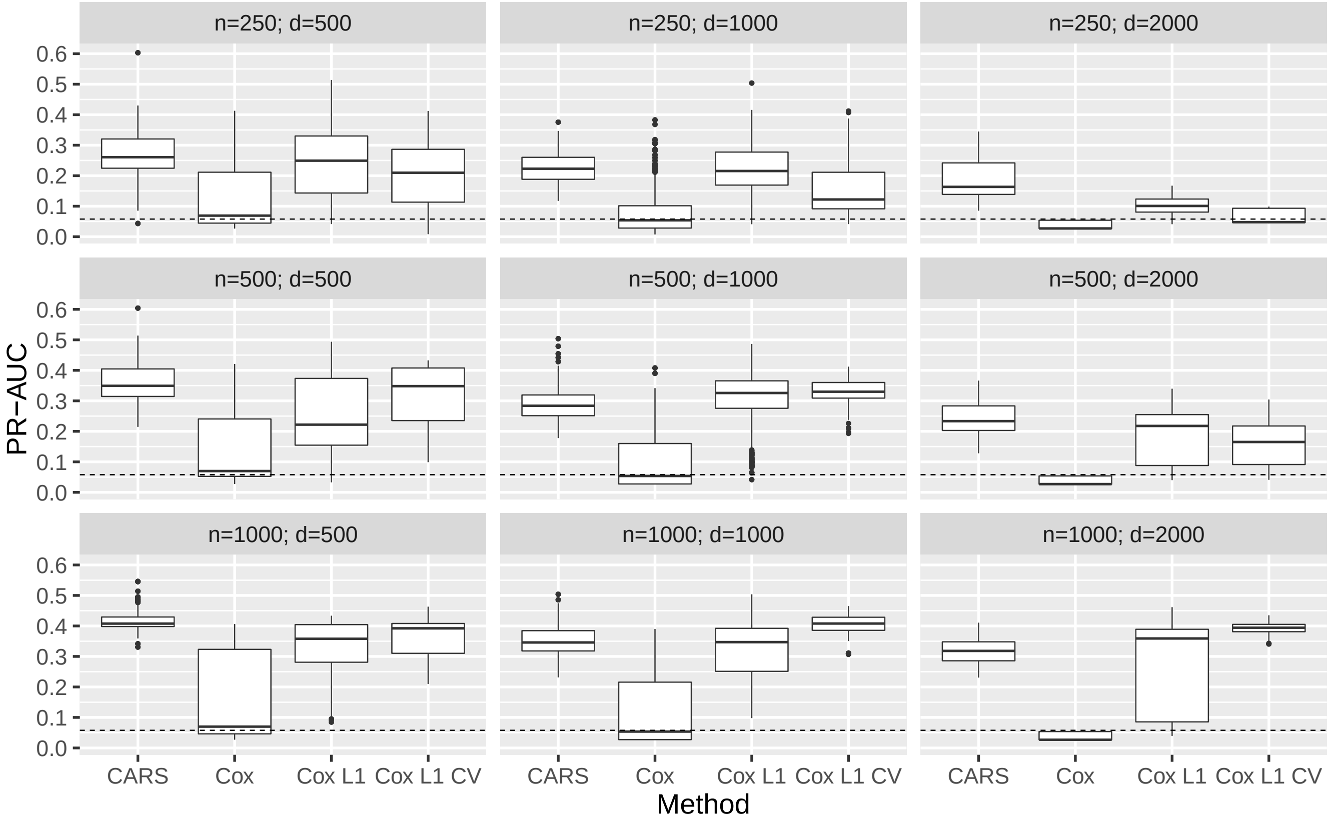


**FIGURE B10** Results of the simulation study. PR-AUC of CARS, Cox and Cox $L_1$ scores stratified by sample size ($n$) and number of covariates ($d$) with low absolute covariate correlations ($\rho = \pm 0.25$) and low censoring rate of 25%. Each boxplot summarizes the results of 2700 simulation runs (3 explained variance ratios x 3 signal to noise ratios x 300 repetitions).

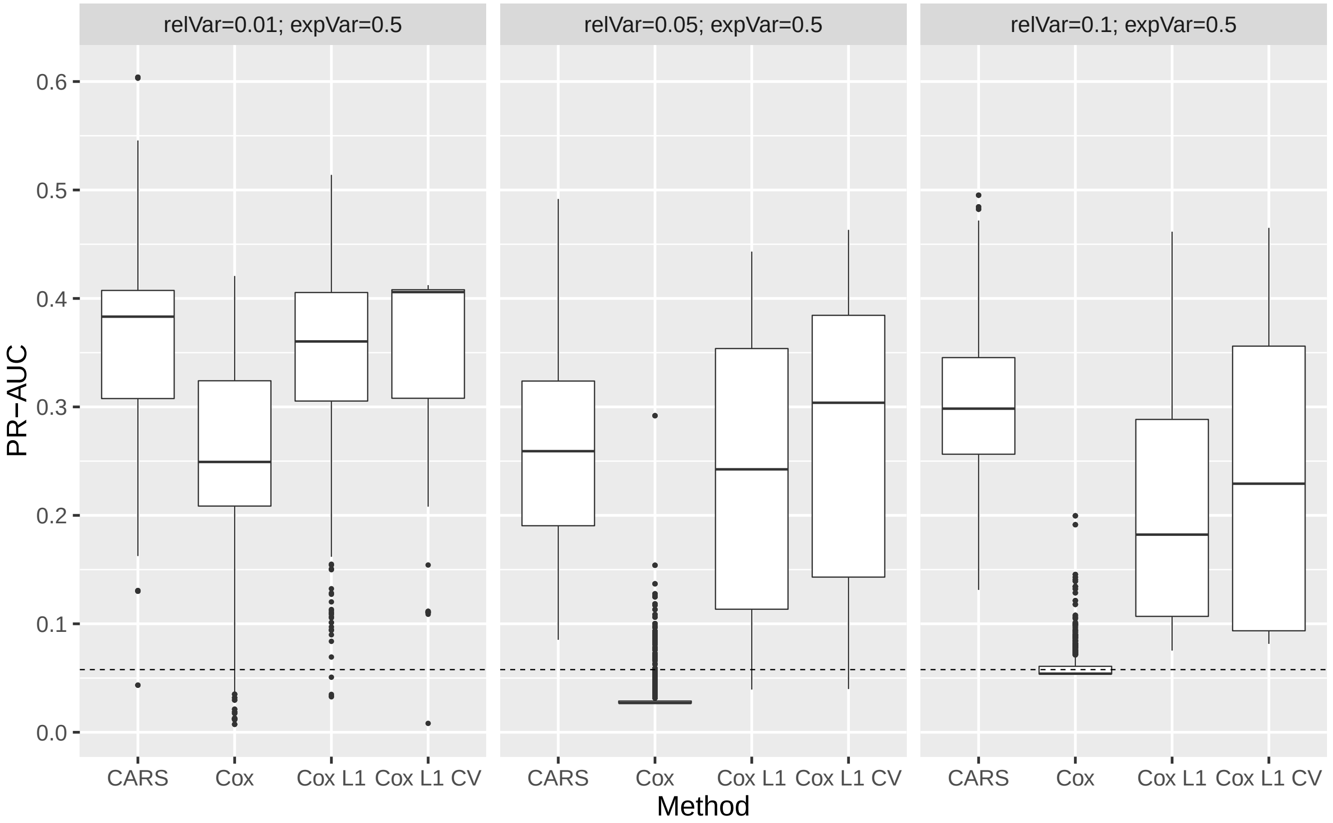


**FIGURE B11** Results of the simulation study. PR-AUC of CARS, Cox and Cox $L_1$ scores stratified by relative number of relevant variables (relVar) and explained variance (expVar) with low absolute covariate correlations ($\rho = \pm 0.25$) and censoring rate of 25%. Each boxplot summarizes the results of 2700 simulation runs (3 sample sizes x 3 number of covariates x 300 repetitions).

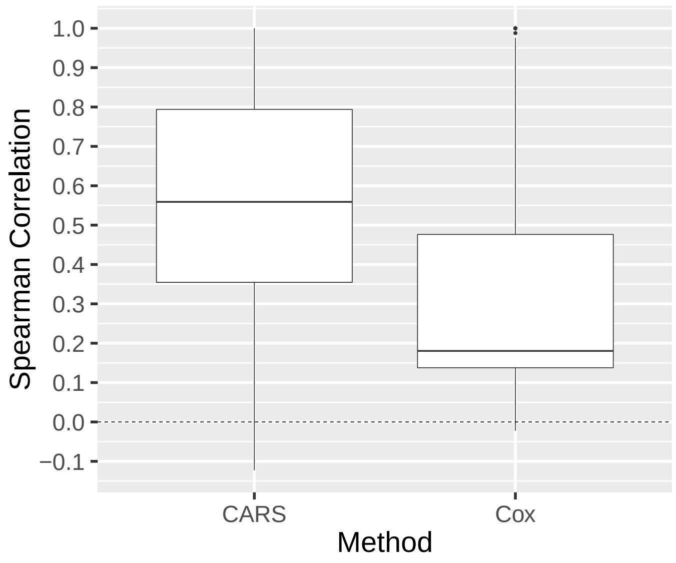


**FIGURE B12** Results of the simulation study, scenario with low absolute correlations ($\rho = \pm 0.25$). The boxplots visualize the rank correlations of the estimated and the true covariate orderings, as obtained from variable selection by CARS and Cox scores. The censoring rate was equal to 25%. Each boxplot shows the results of 24300 simulation runs (3 explained variance ratios x 3 signal to noise ratios x 3 sample sizes x 3 number of covariates x 300 repetitions).

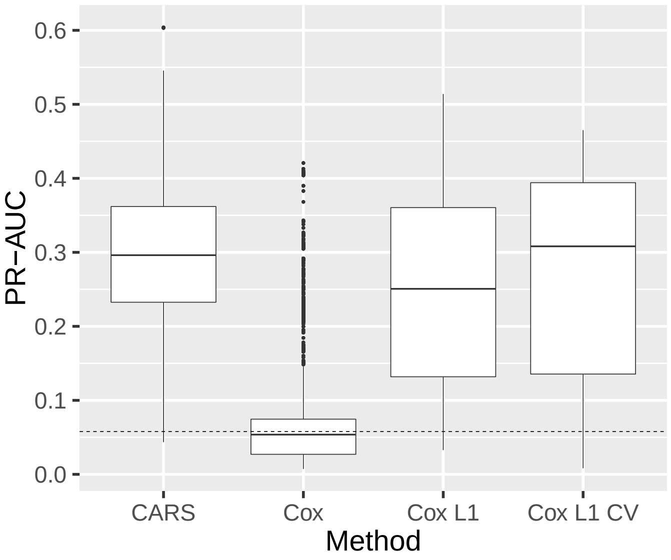


**FIGURE B13** Results of the simulation study, scenario with low absolute correlations ($\rho = \pm 0.25$). The boxplots visualize the PR-AUC values obtained from variable selection by CARS scores, Cox scores and $L_1$-penalized Cox regression. The censoring rate was equal to 25%. The average prevalance of the positive class (computed from all simulations) is displayed by the dashed line. Note that the boxplots contain the PR-AUC values corresponding to all three rates of influential covariates (1%, 5%, 10%). Each boxplot shows the results of 24300 simulation runs (3 explained variance ratios x 3 signal to noise ratios x 3 sample sizes x 3 number of covariates x 300 repetitions).

## B.5 CARS simulation with high absolute covariate correlations and Weibull distribution

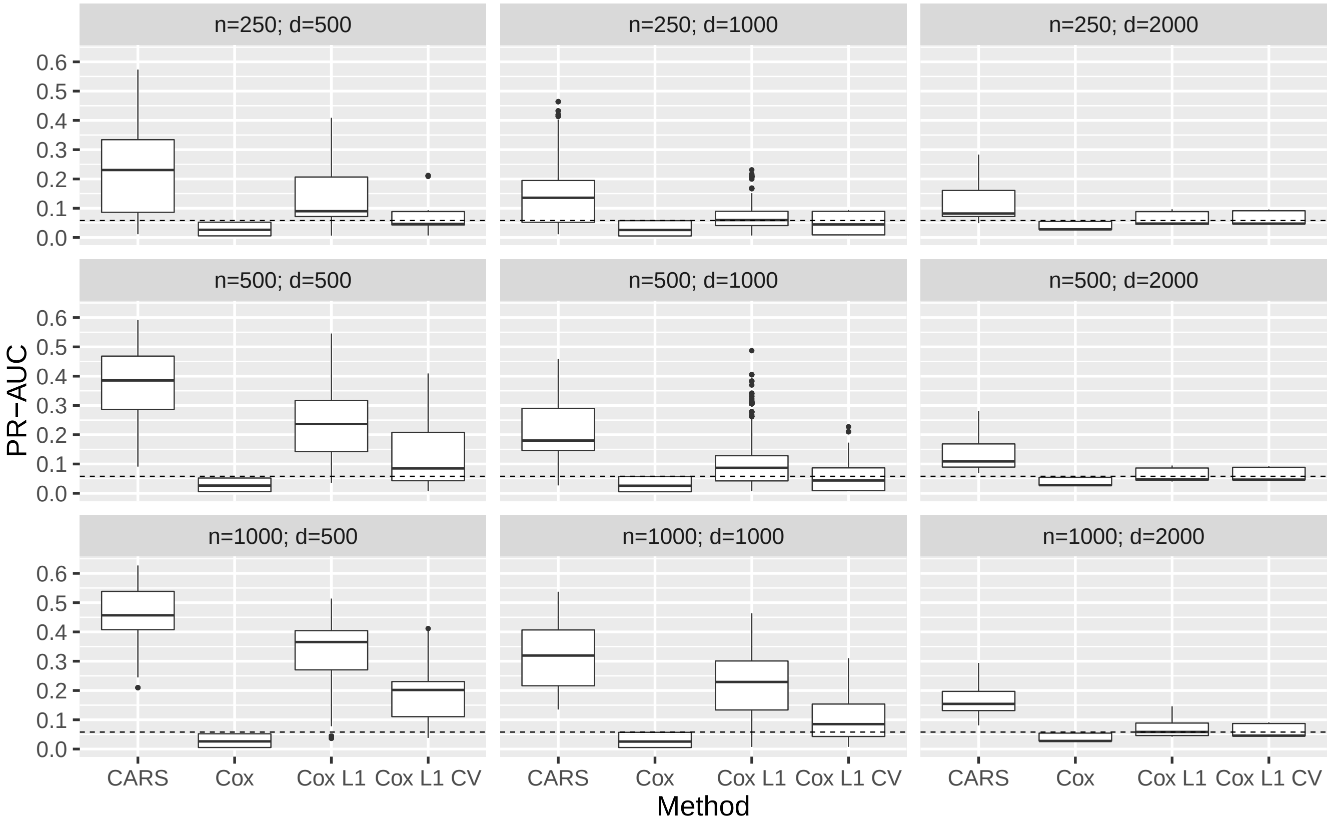


**FIGURE B14** Results of the simulation study. PR-AUC of CARS, Cox and Cox $L_1$ scores stratified by sample size ($n$) and number of covariates ($d$) with high absolute covariate correlations $\rho = \pm 0.75$ and censoring rate of 25%. Each boxplot shows the results of 2700 simulation runs (3 explained variance ratios x 3 signal to noise ratios x 300 repetitions).

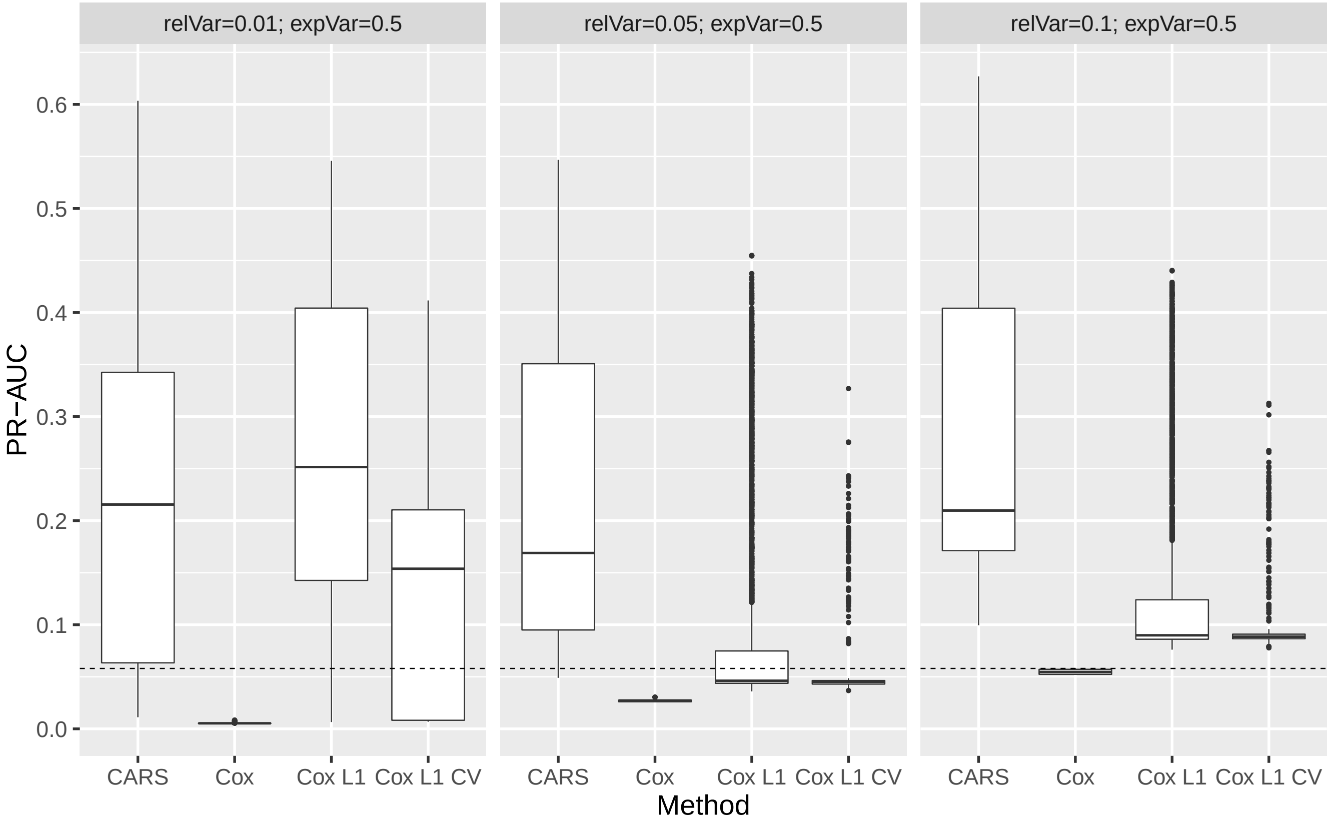


**FIGURE B15** Results of the simulation study. PR-AUC of CARS, Cox and Cox $L_1$ scores stratified by relative number of relevant variables (relVar) and explained variance (expVar) with high absolute covariate correlations $\rho = \pm 0.75$ and censoring rate of 25%. Each boxplot shows the results of 2700 simulation runs (3 sample sizes x 3 number of covariates x 300 repetitions).

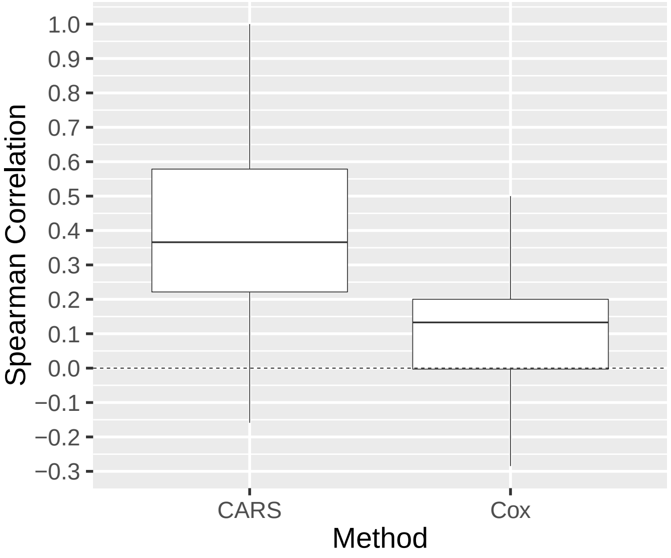


**FIGURE B16** Results of the simulation study, scenario with high absolute correlations ($\rho = \pm 0.75$). The boxplots visualize the rank correlations of the estimated and the true covariate orderings, as obtained from variable selection by CARS and Cox scores. The censoring rate was equal to 25%. Each boxplot shows the results of 24300 simulation runs (3 explained variance ratios x 3 signal to noise ratios x 3 sample sizes x 3 number of covariates x 300 repetitions).

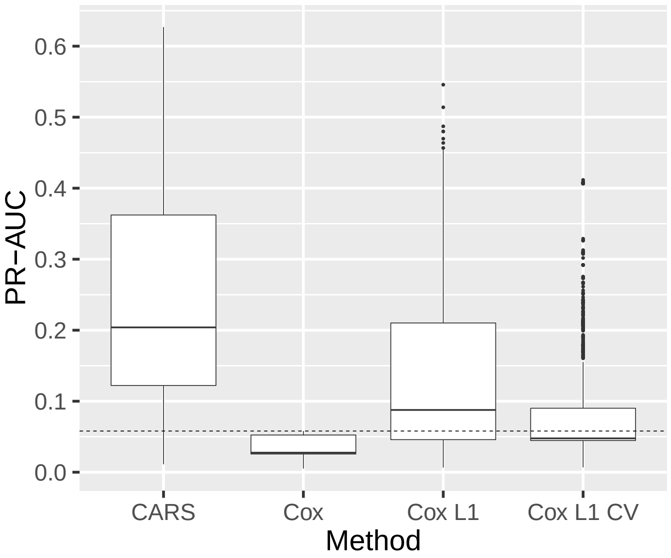


**FIGURE B17** Results of the simulation study, scenario with high absolute correlations ($\rho = \pm 0.75$): The boxplots visualize the PR-AUC values obtained from variable selection by CARS scores, Cox scores and $L_1$-penalized Cox regression. The censoring rate was equal to 25%. The average prevalance of the positive class (computed from all simulations) is displayed by the dashed line. Note that the boxplots contain the PR-AUC values corresponding to all three rates of influential covariates (1%, 5%, 10%). Each boxplot shows the results of 24300 simulation runs (3 explained variance ratios x 3 signal to noise ratios x 3 sample sizes x 3 number of covariates x 300 repetitions). Note that all PR-AUC values of the Cox score approach are below the PR-AUC of a random classifier.

## B.6 Runtime in the low correlation, low censoring scenario

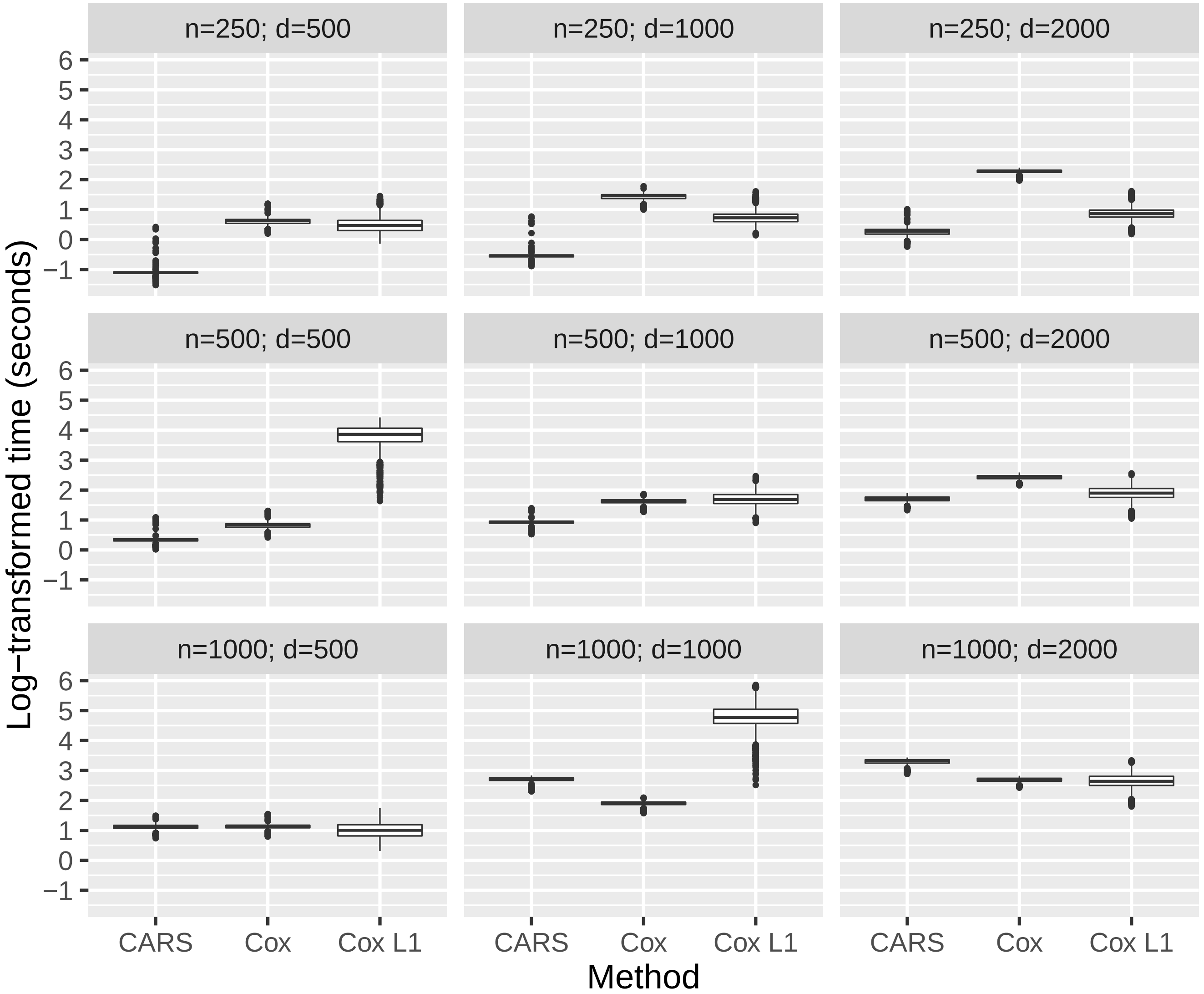


**FIGURE B18** Simulation study on computational efficiency. The boxplots show the log-transformed run-times (in seconds) of the three methods (variable selection by CARS scores, Cox scores and $L_1$-penalized Cox regression). Run-times were measured without parallelization. Each boxplot shows the results of 2700 simulation runs (3 explained variance ratios x 3 signal to noise ratios x 300 repetitions).

## B.7 Construction of correlation matrix

In this section we provide more details on the construction of the correlation matrices used in all scenarios of the simulation (see Section 3.1). First we created a preparatory design matrix $\boldsymbol{B}$ whose diagonal consisted of three equally sized blocks $\boldsymbol{A}_{\xi}$, with $\xi = \{0.25, 0.5, 0.75\}$ referring to the desired absolute correlations. Within each block, half of the correlations were positive and the other half were negative. The first half of positive $\xi$ correlations were inserted columwise into the upper triangle part of the block matrix $\boldsymbol{A}_{\xi}$. The remaining empty entries of the upper triangle part of $\boldsymbol{A}_{\xi}$ were filled with the negative $\xi$ correlations. For example consider the case with 12 covariates: Then each block matrix $\boldsymbol{A}_{\xi} \in \mathbb{R}^{4\times 4}$ would be given by the matrix

$$\boldsymbol{A}_{\xi} = \begin{pmatrix} 1 & \xi & \xi & -\xi \\ \xi & 1 & \xi & -\xi \\ \xi & \xi & 1 & -\xi \\ -\xi & -\xi & -\xi & 1 \end{pmatrix} \tag{B30}$$

In the respective block diagonal matrix $\boldsymbol{B}$, all correlations between variables belonging to different blocks were set to zero. Then the matrix $\boldsymbol{B}$ was converted to the nearest possible positive definite matrix $\tilde{\boldsymbol{B}}$, as measured by a weighted Frobenius Norm of the elementwise differences between the specified and the new matrix. For further details on the algorithm to minimize the deviations between $\tilde{\boldsymbol{B}}$ and $\boldsymbol{B}$ we refer to Higham (2002).[6] A histogram with relative frequencies of the correlation matrix entries, after applying the algorithm by Higham, above the diagonal based on 1000 covariates is shown in Figure B19.

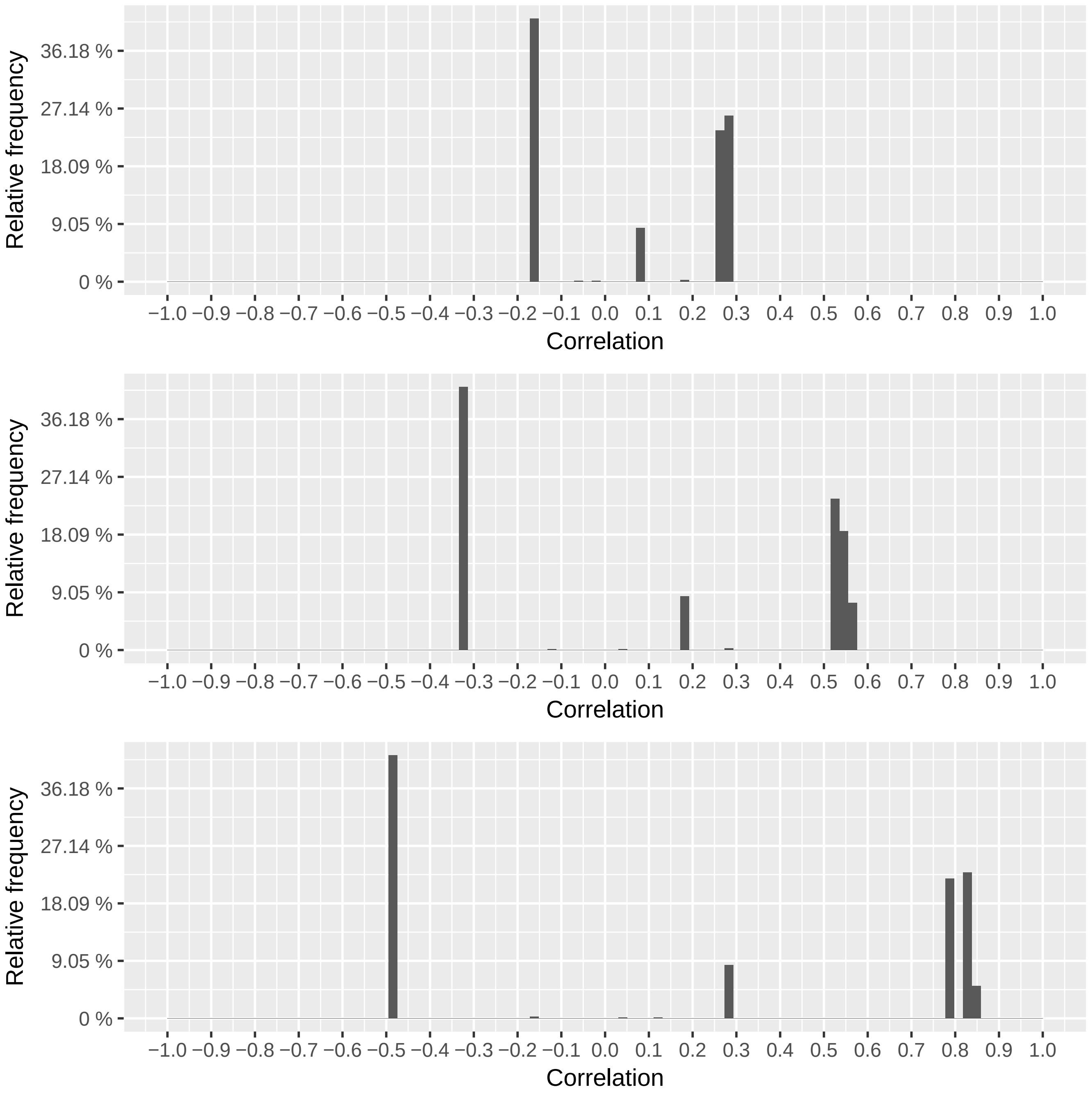


**FIGURE B19** Construction of correlation matrices for the simulation study. The top figure shows the histogram (100 equally sized intervals) of the first block of the final correlation matrix with 1000 covariates. Only correlations above the diagonal are shown. The middle figure shows the histogram of the second block and the lower figure gives the relative frequencies of the third block.

# C ADDITIONAL MATERIAL USED FOR THE DATA ANALYSIS

## C.1 CARS scores: Selected variables in data analysis

| Gene symbol | CARS score | Q-value |
|---|---|---|
| NM_007244 | 0.1063 | 0.0461 |
| NM_004912 | -0.1035 | 0.0461 |
| NM_203281 | -0.1016 | 0.0461 |
| NM_001012271 | -0.0944 | 0.0814 |
| NM_021992 | -0.0939 | 0.0835 |
| NM_006818 | 0.0939 | 0.0836 |
| NM_005722 | -0.0928 | 0.0877 |
| NM_003855 | -0.0923 | 0.0895 |
| NM_001186 | -0.0916 | 0.0918 |
| NM_006846 | 0.0906 | 0.0965 |

**TABLE C1** Prostate cancer application. CARS scores and q-values below $\alpha = 0.1$ ordered by absolute score magnitude. Gene symbol refers to the accession number in the NCBI database.[7]

| Gene Symbol | CARS score | Q-value |
|---|---|---|
| GRIN2C | -0.0479 | $1.0560 * 10^{-6}$ |
| RGP1 | 0.0446 | $6.8075 * 10^{-6}$ |
| TSPAN5 | 0.0446 | $6.8776 * 10^{-6}$ |
| RGS12 | 0.0441 | $7.7700 * 10^{-6}$ |
| ITGA2B | 0.0404 | $1.1334 * 10^{-4}$ |
| SURF1 | -0.0402 | $1.3162 * 10^{-4}$ |
| ZC2HC1A | 0.0386 | $3.7071 * 10^{-4}$ |
| MCM9 | 0.0369 | $1.0399 * 10^{-3}$ |
| TRRAP | 0.0368 | $1.0732 * 10^{-3}$ |
| SLC4A5 | 0.0360 | $1.7160 * 10^{-3}$ |
| FEZ2 | -0.0356 | $2.1184 * 10^{-3}$ |
| ARPC4 /// ARPC4-TTLL3 /// TTLL3 | 0.0347 | $3.2360 * 10^{-3}$ |
| KERA | 0.0347 | $3.3192 * 10^{-3}$ |
| GPR98 | 0.0341 | $4.4523 * 10^{-3}$ |
| SEPT6 | 0.0336 | $5.6147 * 10^{-3}$ |
| PRUNE2 | 0.0334 | $6.1200 * 10^{-3}$ |
| PLEKHG3 | 0.0332 | $6.6953 * 10^{-3}$ |
| AW973834[1] | 0.0329 | $7.4615 * 10^{-3}$ |
| CYP2C8 | 0.0326 | $8.2900 * 10^{-3}$ |
| CUZD1 | 0.0326 | $8.4730 * 10^{-3}$ |
| CTSF | 0.0324 | $8.8681 * 10^{-3}$ |
| KIAA0485 | 0.0323 | $9.0860 * 10^{-3}$ |

**TABLE C2** Breast cancer application. CARS scores and q-values below $\alpha = 0.01$ ordered by absolute score magnitude. Gene symbol refers to the accession number in the NCBI database[7] or proteins.

## C.2 Summary of gene enrichment analysis

| GO.ID | Term | Ann | Signif | Expect | p-value |
|---|---|---|---|---|---|
| GO:0070735 | Protein-glycine ligase activity | 2 | 1 | 0 | 0.0020 |
| GO:0070736 | Protein-glycine ligase activity | 2 | 1 | 0 | 0.0020 |
| GO:0005088 | Ras guanyl-nucleotide exchange factor | 373 | 3 | 0.35 | 0.0057 |
| GO:0008510 | Sodium:bicarbonate symporter activity | 10 | 1 | 0.01 | 0.0099 |
| GO:0101020 | Estrogen 16-alpha-hydroxylase activity | 10 | 1 | 0.01 | 0.0099 |
| GO:0005085 | Guanyl-nucleotide exchange factor activity | 470 | 3 | 0.44 | 0.0107 |
| GO:0004972 | NMDA glutamate receptor activity | 13 | 1 | 0.01 | 0.0128 |
| GO:0070051 | Fibrinogen binding | 13 | 1 | 0.01 | 0.0128 |
| GO:0033695 | Oxidoreductase activity acting on CH | 15 | 1 | 0.01 | 0.0148 |
| GO:0034875 | Caffeine oxidase activity | 15 | 1 | 0.01 | 0.0148 |
| GO:0016881 | Acid-amino acid ligase activity | 21 | 1 | 0.02 | 0.0207 |
| GO:0005452 | Inorganic anion exchanger activity | 22 | 1 | 0.02 | 0.0217 |
| GO:0015106 | Bicarbonate transmembrane transporter activity | 23 | 1 | 0.02 | 0.0226 |
| GO:0016725 | Oxidoreductase activity, acting on CH | 23 | 1 | 0.02 | 0.0226 |
| GO:0015301 | Anion:anion antiporter activity | 26 | 1 | 0.02 | 0.0255 |
| GO:0004970 | Ionotropic glutamate receptor activity | 29 | 1 | 0.03 | 0.0284 |
| GO:0005234 | Extracellular-glutamate-gated ion channel | 30 | 1 | 0.03 | 0.0294 |
| GO:0008324 | Cation transmembrane transporter activity | 703 | 3 | 0.66 | 0.0309 |
| GO:0004129 | Cytochrome-c oxidase activity | 33 | 1 | 0.03 | 0.0323 |
| GO:0015002 | Heme-copper terminal oxidase activity | 33 | 1 | 0.03 | 0.0323 |
| GO:0016676 | Oxidoreductase activity | 33 | 1 | 0.03 | 0.0323 |
| GO:0008391 | Arachidonic acid monooxygenase activity | 34 | 1 | 0.03 | 0.0333 |
| GO:0008392 | Arachidonic acid epoxygenase activity | 34 | 1 | 0.03 | 0.0333 |
| GO:0016675 | Oxidoreductase activity | 34 | 1 | 0.03 | 0.0333 |
| GO:0070330 | Aromatase activity | 34 | 1 | 0.03 | 0.0333 |
| GO:0017112 | Rab guanyl-nucleotide exchange factor activity | 39 | 1 | 0.04 | 0.0381 |
| GO:0008066 | Glutamate receptor activity | 42 | 1 | 0.04 | 0.0409 |
| GO:0016712 | Oxidoreductase activity | 42 | 1 | 0.04 | 0.0409 |
| GO:0022824 | Transmitter-gated ion channel activity | 44 | 1 | 0.04 | 0.0429 |
| GO:0022835 | Transmitter-gated channel activity | 44 | 1 | 0.04 | 0.0429 |
| GO:0015296 | Anion:cation symporter activity | 46 | 1 | 0.04 | 0.0448 |
| GO:0008395 | Steroid hydroxylase activity | 50 | 1 | 0.05 | 0.0486 |

**TABLE C3** Breast cancer application. Gene enrichment analyis based on Fisher's test with *R* package *topGO*. The first column gives the gene ontology (GO)[8] identification number and column "Term" gives additional details. The number in column "Ann" shows how many genes are annotated with the GO term. Column "Signif" gives the number of significant genes of the GO term with respect to q-value 0.05. "Expect" shows the expected number of significant genes under the null hypothesis, e.g. no genes are enriched. The last column "p-value" gives the p-value for Fisher's test for enrichment.

## C.3 CARS scores diagnostic plots

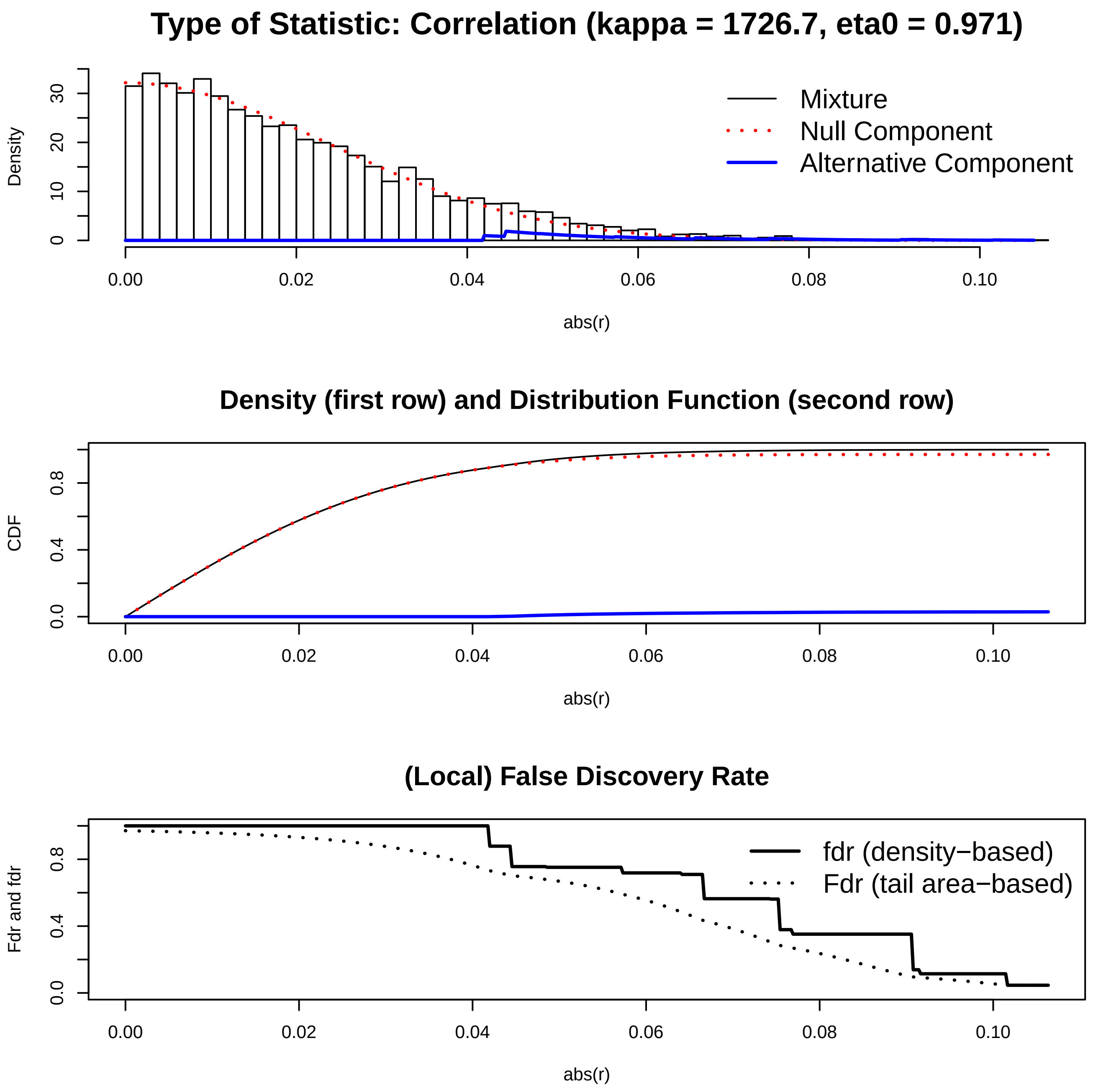


**FIGURE C20** Diagnostic plots obtained in the prostate cancer application (density of empirical null model, cumulative distribution function and local FDR). The first two plots show that the mixture model fits the empirical distribution well.

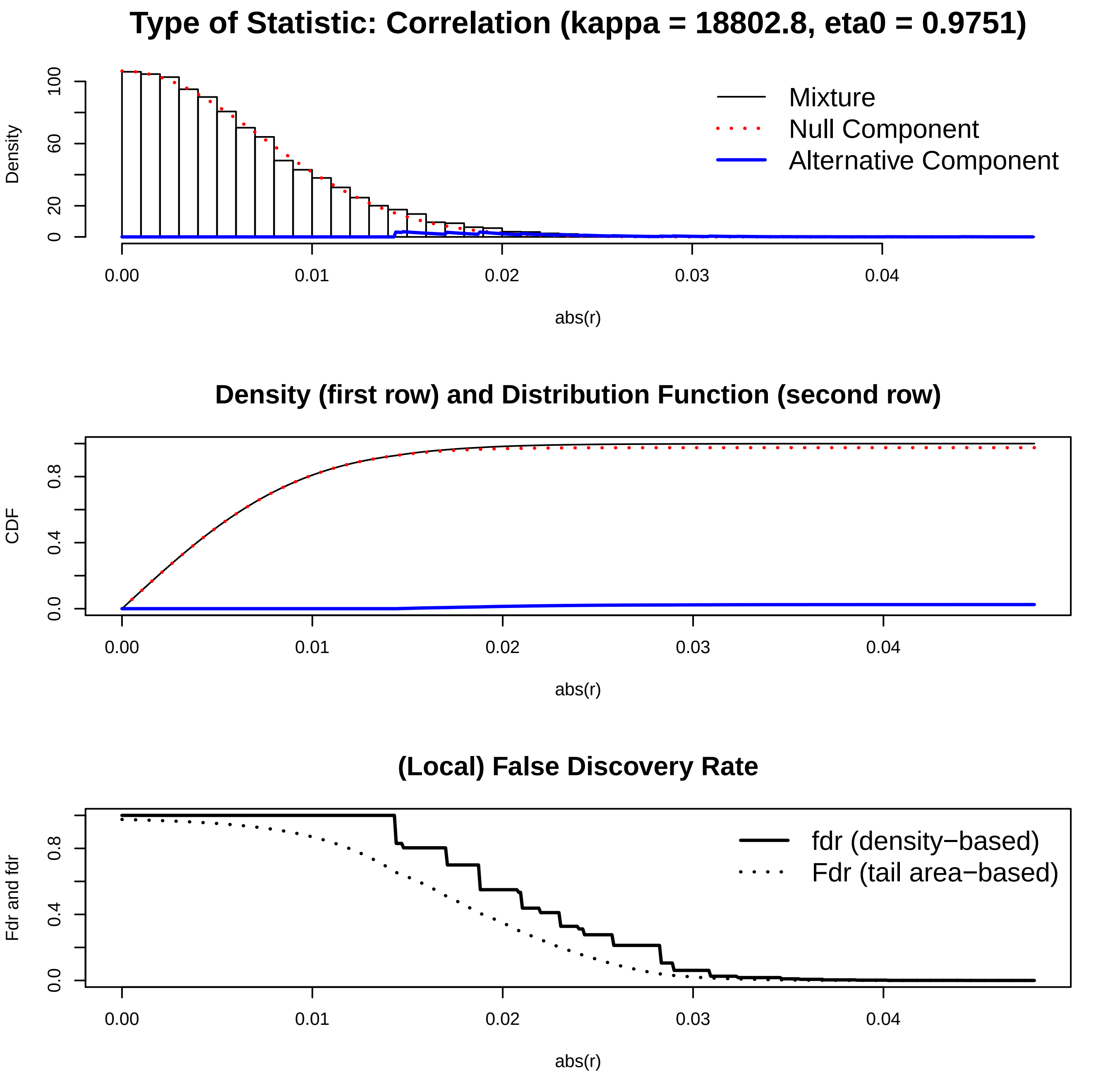


**FIGURE C21** Diagnostic plots obtained in the breast cancer application (density of empirical null model, cumulative distribution function and local FDR). The first two plots show that the mixture model fits the empirical distribution well.

## References


1. Huber P J. The Behaviour Under Maximum Likelihood Estimates Under Nonstandard Conditions. In: Proceedings of the Berkeley Symposium on Mathematical Statistics and Probability; 1967.

2. Carroll R J, Ruppert D, Stefanski L A, et al . *Measurement Error in Nonlinear Models: A Modern Perspective*. New York: Chapman & Hall/CRC; 2 ed.2006.

3. Brookhart M A, Schneeweiss S, Rothman K J, et al . Variable Selection for Propensity Score Models. *American Journal of Epidemiology.* 2006;163(12):1149–1156.

4. Schneeweiss S, Rassen J A, Glynn R J, et al . High-Dimensional Propensity score Adjustment in Studies of Treatment Effects using Health Care Claims Data. *Epidemiology.* 2009;20(4):512–522.

5. Schäfer J, Strimmer K. A Shrinkage Approach to Large-Scale Covariance Matrix Estimation and Implications for Functional Genomics. *Statistical Applications in Genetics and Molecular Biology.* 2005;4(1):1-30.

6. Higham N J. Computing the Nearest Correlation Matrix: A Problem from Finance. *IMA Journal of Numerical Analysis.* 2002;22(3):329-343.

7. Edgar R., Domrachev M., Lash A. E.. Gene Expression Omnibus: NCBI gene expression and Hybridization Array Data Repository. *Nucleic Acids Research.* 2002;30(1):207-210.

8. Consortium The Gene Ontology. Creating the Gene Ontology Resource: Design and Implementation. *Genome Research.* 2001;11(8):1425-1433.
